# Genetic Biomarkers for Periodontal Diseases: A Systematic Review

**DOI:** 10.1111/jcpe.14149

**Published:** 2025-04-08

**Authors:** H. Dommisch, D. Hoedke, E. M.‐C. Lu, A. Schäfer, G. Richter, J. Kang, L. Nibali

**Affiliations:** ^1^ Department of Periodontology, Oral Medicine and Oral Surgery, Charité – Universitätsmedizin Berlin, Corporate Member of Freie Universität Berlin Humboldt‐Universität zu Berlin, Berlin Institute of Health Berlin Germany; ^2^ Periodontology Unit, Centre for Host–Microbiome Interactions, Faculty of Dentistry, Oral and Craniofacial Sciences King's College London London UK

**Keywords:** genetic test, genome‐wide association studies, polygenic risk score, sensitivity, single nucleotide polymorphism, specificity

## Abstract

**Aims:**

To identify genetic biomarkers that may be used in the diagnosis, prevention or management of different forms of periodontal disease.

**Materials and Methods:**

Following protocol registration and PICOTS (patient, intervention, comparison, outcome, time, studies) questions, a systematic search of the literature was conducted (PudMed, Ovid), resulting in 1592 papers screened by two reviewers. Diagnostic data were extracted or calculated from included papers and compared with clinically determined diagnoses, disease progression and/or response to treatment.

**Results:**

A total of 607 articles met the inclusion criteria, including 10 reporting data on gingivitis and 597 on periodontitis. Only two papers reported diagnostic performance data, while for 41 articles on large candidate gene studies, diagnostic performance could be calculated from the reported data. No study using chair‐side tests was identified. Low to moderate values for sensitivity, specificity, positive and negative predictive value and diagnostic accuracy were found.

**Conclusion:**

No genetic diagnostic test of clinical value emerged for periodontal diagnosis, prevention or prediction of disease resolution. Thus, potential future applications of polygenic risk scores that encode susceptibility, as well as single‐marker testing for monogenic or oligogenic forms of periodontal diseases, are discussed.

## 1 Introduction

Periodontitis is a common, complex inflammatory disease belonging to the group of chronic non‐communicable diseases (CNCDs) (Meyle and Chapple 2015; Papapanou et al. 2018). The complexity of the aetiopathogenesis of periodontitis arises as a result of numerous acquired and congenital risk factors, which largely affect immunological competence and metabolism (Meyle and Chapple 2015). The complex interplay between all these factors is still not completely understood, and it is difficult to disentangle the effect of each individual contributing factor in the pathogenesis of periodontitis. In this context, genetic risk factors have been the focus of scientific interest for decades. The pathogenesis of periodontal diseases involves complex interactions between the oral microbiota and the oral mucosal barrier, in conjunction with the host immune system, which are determined by genetic factors and environmental risk factors such as smoking (Genco and Borgnakke 2013). Because of this complexity and the variability of these factors between individuals, periodontitis manifests heterogeneously, including differences in age at onset, severity and progression. The genetic architecture, defined as the overall composition of genetic variants influencing a given trait in combination with external influences such as personal lifestyle, determines the ability of the host's mucosal barrier and immune system to maintain the integrity of the gingiva and thus oral health. Therefore, knowledge of the genetic factors that increase susceptibility to periodontitis has the potential to be used as biomarkers for estimating personal genetic risk (Khera et al. 2018). In the long term, using an individual's genetic profile also holds the potential for guided decisions made about diagnosis, treatment and prevention of disease. This practice of health care emerging from genetic research is termed precision medicine and has the potential to transform our understanding in ways that may allow predictions about disease risk for individual patients that can help choose individual prevention or treatment plans.

GWAS have been particularly useful in finding genetic variations for complex human diseases and traits (https://www.ebi.ac.uk/gwas/home). Several GWAS have been conducted to identify the genetic basis of periodontitis (Buniello et al. 2019). However, the evidence from most studies was limited by small sample sizes and the inclusion of unclear phenotypes, sometimes resulting from self‐reports (Gao et al. 2024), which could lead to misclassification bias. Consequently, despite a large number of candidate‐gene association studies that have demonstrated non‐significant findings, susceptibility genes of periodontal diseases with strong statistical evidence are scarce and only a few genetic risk loci have sufficient statistical evidence to be recognised as genetic risk factors of common forms of periodontitis (Schaefer 2018). These are located at the genes *SIGLEC5*, *DEFA1A3* and *FCER1G*, and those are associated with periodontitis at a genome‐wide significance level (De Almeida et al. 2024; Munz et al. 2018; Shungin et al. 2019). This significance level, typically set at *p* < 5 × 10^−8^, is a critical threshold used to determine whether an observed association between a genetic variant (usually a single nucleotide polymorphism, or SNP) and a trait or disease is statistically significant. A *p*‐value passing the genome‐wide significance threshold provides strong evidence that a particular common SNP is truly associated with the trait of interest and that the result is not due to chance. This threshold is critical because GWAS test millions of SNPs, and without a stringent threshold, random noise could be misinterpreted as true associations. As a rule, common susceptibility variants that are found by GWAS have low effect sizes and play a role in disease phenotypes that develop at adult age. This is because variants with large effect sizes cause severe clinical phenotypes that are rare in the population and work independently of extrinsic risk factors, such as smoking, leading to disease manifestation at a young age (Wray et al. 2018). Therefore, early onset forms of periodontitis may be caused by rare variants with strong effect sizes (Fuchsberger et al. 2016; Genovese et al. 2016). Because of the strong effect sizes of such rare functional variants or mutations, they are qualified targets as genetic markers of increased susceptibility. Reliable criteria to identify a genetic biomarker for a specific disease are further described in Box 1.

BOX 1 **Reliable criteria to identify a genetic biomarker for a specific disease (Aronson and Ferner 2017; Manolio et al. 2009; Xu et al. 2014).**

1

1. The *p*‐value of the association should generally have a genome‐wide significance level (*P* < 5 × 10^−8^) to minimize false positive results. In the case that the *p*‐value has not reached a genome‐wide significance level, for example as a result of limited statistical power, the variant should be significantly associated with the disease in at least two independent studies with a large sample size (ideally several thousand subjects). Ideally, this also applies to genome‐wide significant associations, e.g. those described for rare or singular variants. Their relevance to the disease should also be independently replicated in comparable samples, or the evidence for a true‐positive association should at least be improved by including an additional sample of sufficient size (e.g. in a meta‐analysis that combines the genetic data from several studies).
2. The odds ratio (OR) or relative risk (RR) should be significantly different from 1 and indicate a clinically relevant change in risk, e.g. an OR of 1.5 or higher. Such high ORs generally are found for rare variants or mutations, which cause early‐onset and very severe phenotypes that are usually monogenic or oligogenic.
3. The variant should have a characterized function that has biological plausibility in the pathophysiology of the disease. For example, functional studies could show that the variant alters gene expression substantially (e.g. a 20% reduction), modifies protein structure or influences other cellular processes. For monogenetic diseases, the mutation could be a known pathogenic change, such as a nonsense mutation or a frameshift that severely impairs or completely inactivates the protein.
4. The biomarker should have a high specificity, ideally over 90%, which means that in over 90% of cases it correctly indicates that a person has the disease and not another disease. For example, a genetic variant could be associated with periodontitis, but not with metabolic, immunological diseases or syndromes.
5. The biomarker should have a sensitivity of at least 80%, so that 80% of people with the disease are correctly identified by the biomarker. In practice, this means that at least 80 out of 100 people with the disease are correctly recognized as having the disease by the biomarker.
6. The biomarker should be proven to improve the diagnosis, prognosis or treatment decision. An example could be a genetic variant that predicts response to a particular therapy, e.g. a variant that indicates a 50% higher response to a particular drug. Another clinically relevant point could be that the biomarker is associated with an early stage of the disease and thus enables early intervention.
7. The test methods should have a high level of accuracy (e.g. over 99%) and reproducibility and be able to be carried out in certified laboratories and the cost per test should be low enough to be used in everyday clinical practice.

Thus, the aim of this systematic review was to identify genetic diagnostic tests in relation to gingivitis and periodontitis detection, progression and resolution/treatment success.

## 2 Materials and Methods

The objective of this systematic review was to identify genetic biomarkers that may be used in the diagnosis, prevention or management of different forms of periodontal disease (i.e., gingivitis and periodontitis). In the absence of a sufficient number of diagnostic studies in the literature, we aimed to (i) discuss genetic biomarkers that show the strongest association with different forms of periodontal disease and (ii) provide guidance on the type of future studies required to ultimately establish these biomarkers as diagnostic aids. In addition, an effort was made to investigate polygenic diseases with a strong environmental component that are similar to periodontitis and see what can be learned from them with respect to genetic biomarkers as diagnostic aids.

### 2.1 Focused Review Questions

The following focused questions were phrased:

1. In dentate individuals with or without periodontal disease, are there genetic diagnostic tests available to distinguish between periodontal health and disease?
2. In dentate individuals with or without periodontitis, can genetic diagnostic tests discriminate between disease progression and stability?
3. In dentate patients with a history of periodontal therapy, can genetic diagnostic tests be applied for the prediction of disease resolution?

### 2.2 Methodology

The PRISMA (Preferred Reporting Items for Systematic Review and Meta‐Analyses) checklist was followed for planning and reporting this systematic review (Liberati et al. 2009; Moher et al. 2009) (Supplemental material 1 of Supporting Information). The authors reviewed and agreed on the protocol, which was subsequently registered in the PROSPERO database (ID 577805, https://www.crd.york.ac.uk/prospero/).

Table 1 shows the PICOTS (patient, intervention, comparison, outcome, time, studies) to answer the focused questions.

**TABLE 1:** Questions on patients, intervention, comparison, outcome, time and studies (PICOTS).

	PICOTS#1	PICOTS#2	PICOTS#3
	Distinction between case categories	Identification of periodontitis progression over time	Prediction of disease resolution treatment success
Patients	Dentate individuals with or without periodontal disease	Dentate individuals with or without periodontitis	Dentate patients with history of periodontal therapy
Intervention	Genetic diagnostic test for the discrimination between Health and gingivitis Health and periodontitis Gingivitis and periodontitis Different stages of periodontitis Different grades of periodontitis	Genetic diagnostic test for the discrimination between disease progression (e.g., ongoing CAL or ABL) and stability	Genetic diagnostic test for the prediction of disease resolution (e.g., pocket closure)
Comparison	Standard clinical or radiographic measures		
Outcome	Diagnostic accuracy (sensitivity, specificity, positive/negative predictive value, area under ROC curve)		
Time	Cross‐sectional	Longitudinal (no time limit)	Longitudinal (no time limit)
Studies	Case–control/cross‐sectional studies (diagnostic accuracy studies)	Longitudinal/randomised controlled trials (reporting prognostic accuracy)	Longitudinal/randomised controlled trials (reporting prognostic accuracy)

Abbreviations: ABL, alveolar bone loss; CAL, clinical attachment level.

### 2.3 Eligibility Criteria

#### 2.3.1 Inclusion Criteria

- Study population:
  ○ dentate individuals with clinical and/or radiographic measures of periodontal disease/periodontal treatment response
  ○ ethnicity and/or population details reported
  ○ control phenotype defined (if controls were included)
  ○ non‐syndromic cases included.
- Presenting genetic tests compared with ‘gold‐standard’ diagnoses based on clinical and/or radiographic criteria.
- Reporting diagnostic accuracy values or prediction estimates related to progression or response to treatment (or diagnostic accuracy values could be calculated from the text or tables).

#### 2.3.2 Exclusion Criteria

- Genome‐wide association studies
- Epigenetic studies
- Self‐reported periodontitis
- < 10 individuals included.

### 2.4 Search Strategy

To specifically address PICOTS#1, #2 and #3, a literature search was performed on electronic databases, including

- PubMed in Medline (https://nobility.nem.nih.gov/pubmed) and
- Ovid in EMBASE (https://ovidsp.dc2.ovid.com).

Results from 1960 to 30 June 2024 were evaluated. For a structured literature search, MeSH terms and free text word combinations were applied. Hand search included a complete search of the *Journal of Clinical Periodontology*, the *Journal of Periodontology*, the *Journal of Periodontal Research* and the *Journal of Dental Research* from January 2000 to 30 June 2024, as well as a search of the reference list of included papers and relevant reviews in the field. Search criteria are provided in Supplemental material 1 of Supporting Information.

Following the hand search, electronic searches were performed in order to identify candidate gene studies not restricted to diagnostic testing (search criteria: Supplemental material 2 of Supporting Information) as well as to specifically identify candidate gene studies aiming to validate gene variants detected in GWAS and including at least 500 participants (at least 250 diagnosed with periodontitis or gingivitis). The aim of the latter additional search was to identify the potential diagnostic value of gene variants initially identified by non‐diagnostic studies (GWAS) (search criteria: Supplemental material 3 of Supporting Information). References of included papers are given by PubMed identification documents (PMIDs) in the corresponding tables.

### 2.5 Data Extraction

Study selection was conducted by two independent reviewers (D.H. and E.M.‐C.L.) in the following stages:

1. Screening potentially suitable titles and abstracts against the inclusion criteria to identify potentially relevant papers, resulting in a complete database by merging studies included by at least one reviewer, and
2. Screening of the full papers identified as possibly relevant from the initial screening.

Hand search and additional electronic search were evaluated by two reviewers (L.N. and H.D.). Studies not meeting the inclusion criteria were excluded. In case of disagreement between reviewers, the decision regarding study eligibility was attempted by consensus. Where continued disagreement was apparent, the decision for study inclusion was made following discussion between reviewers. For the initial systematic search, the level of agreement between reviewers was calculated with *κ*‐statistics for the first‐ and second‐stage screening on results regarding PICOTS#1, #2 and #3.

Items for data extraction are described in Supplemental material 4 of Supporting Information. As many of the papers searched were published prior to the current classification of periodontal diseases, an acceptable definition of periodontitis was considered as follows: at least two non‐adjacent sites in different teeth with attachment loss of at least 3 mm on at least two non‐adjacent teeth (diagnosed by dentist/specialist) based on disease state/severity (stage/grade of periodontitis, aggressive vs. chronic periodontitis) (Tonetti et al. 2005). In cases where this was not reported, the validity of the reported periodontitis diagnosis was assessed against this gold standard.

### 2.6 Diagnostic Accuracy Assessment

Diagnostic (or prognostic) accuracy was assessed, which consisted of a comparison between the diagnostic test being evaluated—referred to as the index test (e.g., a novel diagnostic test for periodontitis)—and a reference standard used to categorise participants with or without the target condition. Diagnostic studies should report (i) a contingency table for binary classification (2 × 2 table based on true positives, true negatives, false positives, false negatives), or (ii) sensitivity and specificity values and sample sizes of the control and target conditions from which the estimation of the classification table was possible (Arias‐Bujanda et al. 2019). For studies reporting diagnostic (or prognostic) accuracy, diagnosis (or prognosis) data were extracted, and the PRISMA‐DTA checklist was used.

### 2.7 Quality Assessment—Risk of Bias

The quality of the included studies was assessed via a risk‐of‐bias analysis, as it may impact the overall results and conclusions. The quality of the included studies was assessed using the critical review checklist of the revised Quality Assessment of Diagnostic Studies (QUADAS‐2) (Whiting et al. 2011). If appropriate, the Newcastle‐Ottawa scale was used for case–control and cohort studies. In cases of candidate gene studies that were not defined as ‘diagnostic’ but reported data to calculate diagnostic values, a specific risk‐of‐bias tool was used (Nibali 2013).

### 2.8 Data Synthesis

A narrative synthesis of the outcomes of included studies is presented. Meta‐analysis of diagnostic accuracy was considered in the presence of a sufficient number of comparable studies with similar design. For diagnostic studies, estimates of accuracy were expressed as sensitivity and specificity values, as well as with 95% confidence intervals (CIs) for each classification of a (epi)genetic biomarker. Accuracy, positive predictive value, negative predictive value, positive likelihood ratio, negative likelihood ratio, area under the curve and diagnostic odds ratio were extracted (or calculated by the reviewers if possible) from each article.

## 3 Results

### 3.1 Study Selection

**FIGURE 1:**
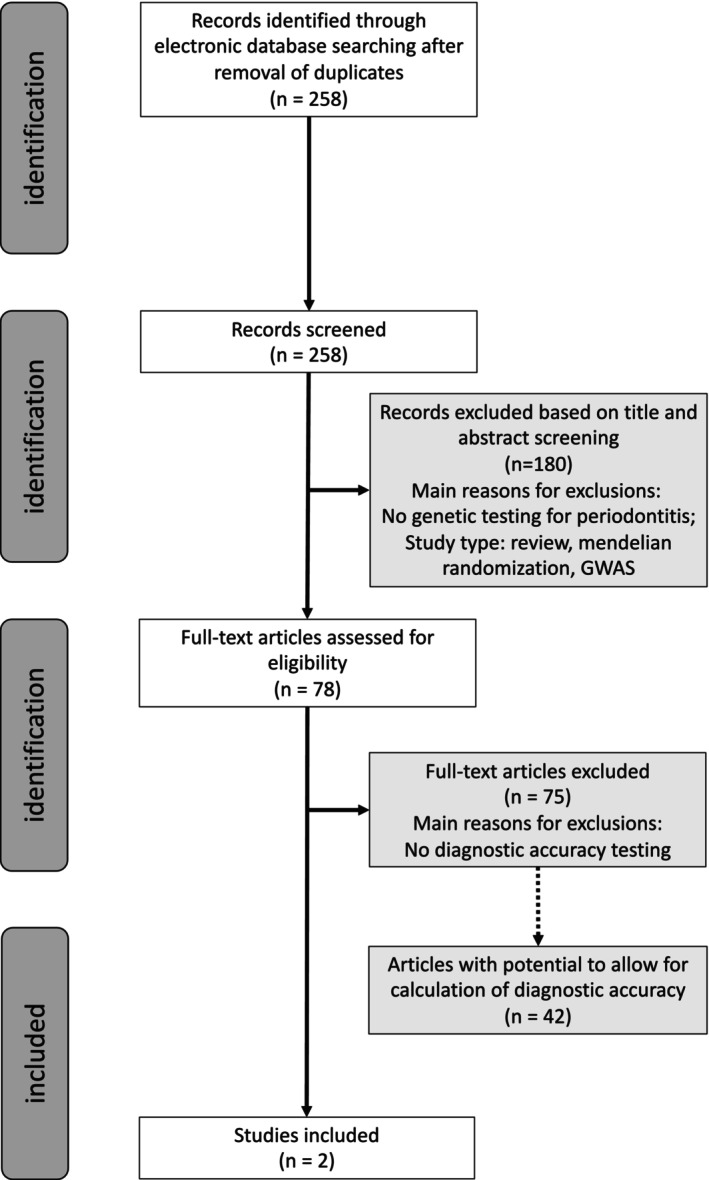
Flow chart of the literature search and screening process.

Figure 1 presents the flow chart of paper inclusion. Electronic search based on PICOTS#1, #2 and #3 resulted in 229 and 207 titles retrieved from PubMed and Ovid search, respectively. After removal of duplicates, a total of 268 papers were included in the first screening. After a first‐stage screening, 78 papers were included in the second screening (*κ* = 0.964). Following a second screening, three papers were found to be suitable and were included. Kappa‐scores were 0.964 and 0.991, respectively, at first and second screening. Reasons for exclusion of papers during the second‐stage screening are presented in Supplemental material 5 of Supporting Information.

Hand and additional electronic search in PubMed resulted in 1592 papers, of which 604 were included in the second‐stage screening, while 597 were finally considered suitable. These include 10 papers on gingivitis (8 for PICOTS#1 and 2 for PICOTS#2, Table 2) and 597 on periodontitis (563 for PICOTS#1 and 24 for PICOTS#2 and #3). A subset of 16 of these papers addressing periodontitis for PICOTS#1 was identified based on including at least 1000, as are outlined in Table 3. An additional 16 papers identified for periodontitis for PICOTS#2 are reported in Table 4 (see also Figure S1). The search on candidate gene studies regarding variants previously identified by GWAS resulted in 745 titles, from which 6 papers were included based on the inclusion criteria (Figure S2).

**TABLE 2:** Parameters of candidate gene studies included in the systematic review based on studies for PICOTS#1 for gingivitis.

Authors, year of publication, PMID	Country, study type	Number of participants	Gingivitis definition	Gene variants	Main results
Studies on gingivitis					
Becerra‐Ruiz et al. 2021 34027804	Mexico Cross‐sectional study	*n* = 171 With gingivitis or periodontal healthy (18–69 years old)	BOP > 10% No periodontal probing depth > 3 mm	TNF‐α (−308 G/A)	TNF‐ α (−308 G/A) carriers of the A/A genotype and allele A: protective towards gingivitis
Barbosa et al. 2020 32902920	Brazil Cohort study	*n* = 353 (8–11 years old)	Gingival bleeding measured by CPI	VDR: rs2228570 (FokI, A>G/Met>Thr) and rs739837 (BglI, G>T)	No genetic influence on gingivitis
Reis et al. 2021 32643487	Brazil Cohort study	*n* = 353 (8–11 years old)	Gingival bleeding measured by CPI	SNPs in *IL‐6* gene (rs1800795) and in *IL‐1β* gene (rs1143627 and rs1143629)	The GG genotype in rs1800795 (*IL‐6*) decreases the risk of gingivitis in a co‐dominant model (*p* = 0.05; OR = 0.64)
Garlet et al. 2012 22324464	Brazil Case–control study	*n* = 411 Cases: *n* = 193 (chronic gingivitis) controls: *n* = 218 (healthy)	High scores of BOP (> 70%) Absent or minimal CAL and bone loss, poor plaque control and no history of tooth loss due to periodontitis	IL‐1β‐3954 (rs1143634), TNFA‐308 (rs1800629), IL‐6‐174 (rs1800795), IL‐10‐592 (rs1800872), TLR4‐299 (rs498670)	rs1800629, rs1800872, rs498670 differentiated between CG and H. rs1143634, rs1800629, rs1800795, rs1800872, rs498670 differentiated between CP and CG.
Vokurka et al. 2009 18930181	Czech Republic Case–control study	*n* = 298 (Caucasians) Cases: *n* = 147 (49.3%) controls: *n* = 151 (51.7%) (11–13 years old)	Modified GI, 6 index teeth, 0–3 scale gingivitis on or adjacent to 4 sites Gingivitis: ≥ 4/72 points Health: < 4/72 points	MMP‐9 (−1562 C/T) (rs3918242) IL‐18 (−607 A/C) (rs1946518)	Only statistically significant association of the composite genotype (MMP‐9 + IL‐18) with gingivitis
Izakovikova et al. 2008 18446552	Czech Republic Case–control study	*n* = 455 (Caucasians) Cases: *n* = 272 Controls: *n* = 183 (249 boys, 206 girls) (11–13 years old)	Modified GI, 6 index teeth, 0–3 scale gingivitis on or adjacent to 4 sites Gingivitis: ≥ 4/72 points Health: < 4/72 points	IL‐6 − 174 G/C (rs1800795) − 572 C/G (rs1800796) − 597 A/G (rs1800797)	IL‐6 174 (rs1800795) G allele was associated with gingivitis versus health
Dashash et al. 2005 16171432	United Kingdom Case–control study	*n* = 260 (Caucasians) Cases: *n* = 174 Controls: *n* = 86 (8–12 years old)	Identified with BOP and GI and divided into: mild (*n* = 93), moderate (*n* = 54), severe (*n* = 27)	IL‐10 −1082 G/A (rs1800896)	IL‐10 rs1800896 A allele was associated with gingivitis (*p* = 0.01)
Müller & Barrieshi‐Nusair 2007 17492470	Kuwait Cross‐sectional study	*n* = 50 (Arabian descent) (19–28 years old)	% of BOP sites 20 s after probing	IL‐1 gene cluster IL‐1A −889 IL‐1B +3954	Genotype positive subjects had fewer BOP sites
Studies on induction of gingivitis					
Jepsen et al. 2003 12622850	Germany Sub‐study from a prospective clinical study investigating untreated areas	*n* = 20 (Caucasians) Genotype +: 10 Genotype −: 10	Experimental gingivitis (21 days) GCF volume BOP (dichotomous)	IL‐1a +4845 and IL‐1ß +3953 genotypes	No associations between IL‐1 genotype on the development of clinical signs of gingivitis
Scapoli et al. 2007 17953695	Italy Randomised split‐mouth localised 21‐day experimental gingivitis clinical trial	*n* = 96 (European Caucasians) (46 males, mean age: 23.9 years and 50 females, mean age: 23.3 years)	Experimental gingivitis. Partial‐mouth GI, PI and angulated bleeding score (AngBS) ‘cumulative plaque exposure’ (CPE)	TNF‐A −308 G/A (rs1800629) LT‐A −252 A/G (rs909253) IL‐6 −597 A/G (rs1800797) IL‐6 −174 G/C (rs1800795)	No associations

Abbreviations: BOP, bleeding on probing; CAL, clinical attachment level; CPI, community periodontal index; G, gingivitis; GCF, gingival crevicular fluid; GI, gingival index; H, healthy.

**TABLE 3:** Parameters of candidate gene studies included in the systematic review based on studies for PICOTS#1 for periodontitis.

Authors, year of publication, PMID	Country, study type	Number of participants	Periodontal diagnosis and definition	Gene variants	Main results	Accuracy data extracted for gene variants with stat sign associations with PD
Asa'ad et al. 2023 37382343	Norway Cross‐sectional study	*n* = 3633 Age: 40–93 years	2017 AAP/EFP classification (no periodontitis, grades A, B or C)	4 SNPs, IDH2, TET1, and TET2 in genes DNMT: – rs2288349 – rs35474715 – rs34023346, and – rs10010325	Age 40–49 years: DNMT1 rs2288349 associated with decreased, while A‐allele of rs10010325 (TET2) with increased susceptibility to periodontitis	— rs2288349 (GG vs. GA + AA): sens 0.35, spec 0.71, acc 0.43 — rs10010325 (AA vs. AC + CC): sens 0.22, spec 0.83, acc 0.36
Yang et al. 2022 35179257	USA Cross‐sectional study	*n* = 1124 (45% Hispanic, 30% Black, 23% White)	CDC/AAP case definition	Various SNPs identified in previous GWAS	IL‐37, HPVC1, TRPS1, ABHD12B, LDLRAD4 (C180rF1), TGM3, and GRK5 associated with %teeth with PD ≥ 4 mm DAB2IP with presence of PD ≥ 6 mm KIAA1715 (LNPK), ROBO2, RAB28, LINC01017, NELL1, LDLRAD4 (C18orF1), and CRYBB2P1 with %teeth with CAL ≥ 3 mm RUNX2 and LAMA2 associated with %teeth were associated with CAL ≥ 5 mm	Not available
She et al. 2021 33854406	China Case–control study	*n* = 1777 Cases: *n* = 884 (CP) Controls: 893 healthy	2017 AAP/EFP classification	– rs1800587 in the promoter of the interleukin‐1*α* gene (−889 C/T)	rs1800587 was significantly associated with CP	rs1800587: sens 0.17, spec 0.88, acc 0.53
Tong et al. 2019 30765789	China Case–control study	*n* = 3160 Cases: *n* = 1076, (CP) controls: *n* = 2084	1999 AAP classification	– NIN – SIGLEC5	SNPs rs12883458 (NIN) and rs4284742 (SIGLEC5) associated with chronic periodontitis rs4284742 (SIGLEC5) with BOP, PD and CAL	— rs12883458 (CC + CT vs. TT): sens 0.22, spec 0.83, acc 0.63 — rs4284742 (GG vs. GA + AA): sens 0.68, spec 0.38, acc 0.49
Folwaczny et al. 2018 28983640	Germany Case–control study	*n* = 1160 Cases: *n* = 390 Controls: *n* = 770	Mild PD: max PPD depth ≤ 6 mm, ≤ 5 teeth RBL > 30%, no AL ≥ 50% at any tooth; Moderate PD: ma PPD ≤ 8 mm, ≤ 8 teeth RBL > 30%, AL ≥ 50% at ≤ 5 teeth; Severe PD: max PPD > 8 mm, > 8 teeth RBL > 30%, AL ≥ 50% at > 5 teeth	SNPs from IRGM gene: – rs13361189 – rs10065172 – rs4958847 – rs1000113, rs11747270, rs931058	Association for rs11747270 with severe periodontitis (*p* = 0.003). Haplotype C‐T‐A‐T‐G significantly associated with chronic periodontitis (*p* = 0.0051; OR 4.66, 95% CI: 1.41–15.42).	rs11747270 (AG + GG vs. AA): sens 0.13, spec 0.89, acc 0.65
Schulz et al. 2018 2973256	Germany Cross‐sectional and longitudinal study	*n* = 1002 (Caucasian CVD patients)	PD: presence of PAL of ≥ 3 mm in ≥ 2 non‐adjacent Severe PD: ≥ 30% of teeth with PAL ≥ 5 mm	SNPs in ANRIL gene – rs1333049 and – rs321799	ANRIL SNPs rs133049 and rs3217992 were not associated with severe periodontitis	Not available
Tanaka et al. 2017 29129846	Japan Cross‐sectional study	*n* = 1148 Cases: *n* = 131 Controls: *n* = 1017 (Post‐partum Japanese women, 18–45 years old)	One tooth with PPD ≥ 4 mm	– rs1946518 (−607 C/A) – rs187238 (−137G/C),	The CC genotype of rs1946518 had a significantly reduced risk of periodontitis	rs1946518 (CA + AA vs. CC): sens 0.90, spec 0.17, acc 0.25
Shang et al. 2015 26643602	China Case–control study	*n* = 5065 Cases: *n* = 1264 Controls: *n* = 3801 (Han Chinese)	≥ 2 interproximal sites with CAL ≥ 6 mm, and ≥ 1 with PPD ≥ 5 mm	65 SNPs in FBXO38, AP3B2, and WHAMM genes	Associations for FBXO38 (rs10043775) and AP3B2 (rs11631963‐rs11637433) and haplotypes with periodontal data	Not available
Wu et al. 2015 24690098	USA Case–control study	Discovery study: *n* = of 880 Caucasians: Cases: *n* = 435 Controls: *n* = 445 *n* = 222 African Americans: Cases: *n* = 151 Controls: *n* = 71; Replication study: *n* = 309 Hispanics: Cases: *n* = 166 Controls: *n* = 143 *n* = 313 Asians: Cases: *n* = 156 Controls: *n* = 157	Various definitions across populations	4 SNPs in IL‐1B gene – rs16944 – rs1143623 – rs4848306 – rs1143633	Association between the IL‐1 genotype pattern and moderate to severe periodontitis across different populations	Not available
Tanaka et al. 2014 24460370	Japan Cross‐sectional study	*n* = 1281 Cases: *n* = 131 Controls: *n* = 1150 Post‐partum Japanese women (18–45 years)	One tooth with PPD ≥ 4 mm	SNPs in IL‐1α (rs1800587) and IL‐1β (rs1143634 and rs16944)	SNP rs16944 GA genotype: reduced risk of periodontitis	rs16944 (AA vs. GA + GG): sens 0.33, spec 0.46, acc 0.48
Zhang et al. 2014 25101955	China Case–control study	*n* = 1150 Cases: *n* = 400 Controls: *n* = 750 (Han Chinese)	1999 AAP classification, with PPD > 5 mm, CAL > 4 mm, gingival recession and mobility	23 SNPs in IL‐8 gene	rs4073 and rs2227307 and haplotypes associated with chronic periodontitis	‘Haplotype 3’ (strongest statistical association): sens 0.10, spec 0.82, acc 0.57
Folwaczny et al. 2011c 21252350	Germany Case–control study	*n* = 1193 Cases: *n* = 402 (CP), controls: *n* = 793 Caucasians (upper Bavarian population)	1999 AAP classification	SNPs in NR1I2 gene: – rs12721602 – rs3814055 – rs1523128 – rs1523127 – rs45610735 – rs6785049 – rs2276707 – rs3814057	The haplotype (GTGAG) composed of rs12721602, rs3814055, rs1523128, rs12721607 and rs6785049 associated with chronic periodontitis	Not available
Folwaczny et al. 2011a 21954916	Germany Case–control study	*n* = 1160 Cases: *n* = 389 (CP), controls: *n* = 771 Caucasians (upper Bavarian population)	1999 AAP classification	– MICA‐TM – MICB‐C 1_2_A – C1_4_1	Significant differences in microsatellite polymorphisms in the MHC class I region between patients and healthy controls	Not available
Folwaczny et al. 2011c 21252350	Germany Case–control study	Cases: *n* = 402 (CP), controls: *n* = 793 Caucasians (upper Bavarian population)	1999 AAP classification	SNPS in PPARG gene: – rs10865710 – rs2067819 – rs3892175 – rs1801282 – rs3856806	No association between PPARG gene variants and chronic periodontitis.	Not available
Schaefer et al. 2010 19829306	Germany Case–control study	*n* = 4224 Cases: *n* = 1337 (AgP or CP) Controls: *n* = 2887 (including 17,91 not reference controls)	1999 AAP classification	Promoter SNPs of DEFB1 gene	rs1047031 was associated with AgP	rs1047031: sens 0.63, spec 0.31, acc 0.41
Schaefer et al. 2009 19214202	Germany Case–control study	*n* = 1392 Cases: *n* = 151 with GAgP; Controls: *n* = 736 controls, cases: *n* = 137 with LAgP Controls: *n* = 368	1999 AAP classification	rs1333048 on human chromosome 9p21.3	rs1333048 was associated with GAgP and LAgP	Not available

Abbreviations: AAP, American Academy of Periodontology; acc, accuracy; CP, chronic periodontitis; CVD, cardiovascular disease; GAgP, generalised aggressive periodontitis; LAgP, localised aggressive periodontitis; PD, periodontitis; PPD, probing pocket depth; sens, sensitivity; SNP, single nucleotide polymorphism; spec, specificity.

**TABLE 4:** Parameters of studies included in the systematic review based on validation of candidate gene studies or replication of genome‐wide association studies for PICOTS#1 for periodontitis.

Authors, year of publication	Country	Number of participants	Periodontal diagnosis and definition	Ethnicity, co‐conditions	Summary of findings
Schaefer et al. 2013	Germany	*n* = 2041 Cases *n* = 600 (AgP) Controls: *n* = 1441	Aggressive periodontitis: less than 35 years old; at least two teeth with > 30% alveolar bone loss, evidenced by radiographs	Caucasian (Turkish and Italian population)	None of the analysed gene was statistically associated with AgP
Taiete et al. 2018	Brazil	*n* = 390 Cases: *n* = 200 (CP) controls: *n* = 190	Aggressive and Chronic periodontitis (1999 AAP classification)	Caucasian (Brazilian population)	SNPs rs6667202 (*IL10*) was detected less frequently in AgP, compared to healthy controls and CP groups
Tong et al. 2019	China	*n* = 3160 Cases: *n* = 1076 (CP) Controls: *n* = 2084	Chronic periodontitis, (1999 AAP classification)	Chinese	SNPs rs12883458 (*NIN*) and rs4284742 (*SIGLEC5*) were significantly associated with chronic periodontitis In particular, rs4284742 (*SIGLEC5*) was significantly associated with BOP, PD and CAL
Cirelli et al. 2021	Brazil	*n* = 714 Cases: *n* = 358 (moderate/severe PD) Controls: *n* = 356 (healthy/mild PD)	Mild, moderate and severe periodontitis, (CDC/AAP case definition)	Caucasian (Brazilian population)	rs2521634‐AA (close to *NPY* gene) showed an increased risk for severe periodontitis rs3811046‐GG (*IL37* gene) showed increased risk for moderate periodontitis
Cirelli et al. 2021	Brazil	*n* = 931 Cases: *n* = 358 (periodontitis): Cases: *n* = 239 (periodontitis and T2DM) Controls: *n* = 334	Moderate or severe periodontitis (CDC/AAP case definition)	Caucasian (Brazilian population) T2DM	rs7957197‐TA (*HNF1A* gene); rs7754840‐CG (*CDKAL1*); rs1531343‐GC (*RPSAP52*); rs7903146‐TT (*TCF7L2*); rs7018475‐GG (*CDKN2B*) The SNPs for *TCF7L2* and *CDKN2B* in female, never smokers, and poorly controlled diabetes type II patients were associated with worse glycaemic condition and periodontal parameters
Yang et al. 2022	USA	Total of 1124 individuals (mix of cases and controls, the numbers for each are not stipulated)	CDC/AAP case definition	45% Hispanic 30% Black 23% White	*CLEC19A* was associated with edentulism and %teeth with pocket depth (PD) ≥ 4 mm *IL37*, *HPVC1*, *TRPS1*, *ABHD12B*, *LDLRAD4* (*C180rF1*), *TGM3* and *GRK5* were associated with %teeth with PD ≥ 4 mm *DAB2IP* with presence of PD ≥ 6 mm *KIAA1715*(*LNPK*), *ROBO2*, *RAB28*, *LINC01017*, *NELL1*, *LDLRAD4*(*C18orF1*) and *CRYBB2P1* were associated with %teeth with clinical attachment level (CAL) ≥ 3 mm *RUNX2* and *LAMA2* were associated with %teeth were associated with CAL ≥ 5 mm KIAA1715(LNPK) was associated with high colonisation by *Aggregatibacter actinomycetemcomitans* *CLEC19A*, *IQSEC1* and *EMR1* associated with microbial abundance based on checkerboard data; LBP and NCR2 were associated with microbial abundance based on sequencing data *NCR2* was associated with microbial diversity based on sequencing data

Abbreviations: ABHD12B; abhydrolase domain containing 12B; AgP, aggressive periodontitis; BOP, bleeding on probing; CAL, clinical attachment level; CDC/AAP, Centres for Disease Control and Prevention/American Academy of Periodontology; CDKAL1, CDK5 regulatory subunit‐associated protein 1‐like 1; CDKN2B, cyclin‐dependent kinase inhibitor 2B; CGS, candidate gene studies; CLEC19A, C‐type lectin domain containing 19A; CRYBB2P1, Crystallin Beta B2 Pseudogene 1DAB2IP, Disabled homologue 2‐interacting protein; EMR1/ADGRE1, adhesion G protein‐coupled receptor E1; GRK5, G protein‐coupled receptor kinase 5; GWAS, genome‐wide association studies; HNF1A, hepatic nuclear factor 1 alpha; HPVC1, human papillomavirus (type 18) E5 central sequence‐like 1; IL‐37, Interleukin 37; IQSEC1, IQ motif and SEC7 domain‐containing protein 1; KIAA1715/LNPK, Lunapark, ER Junction Formation factor; LAMA2, laminin subunit alpha‐2; LBP, lipopolysaccharide binding protein; LDLRAD4, low‐density lipoprotein receptor class A domain containing 4; LINC01017, long intergenic non‐protein coding RNA 1017; NCR2, Natural cytotoxicity triggering receptor 2; NELL1, Neural Epidermal Growth Factor‐Like 1; NIN, ninein; NPY, neuropeptide Y; PD, pocket depths; RAB28, Ras‐related protein Rab‐28; ROBO2, roundabout guidance receptor 2; RPSAP52, ribosomal protein SA pseudogene 52; RUNX2, Runt‐Related Transcription Factor 2; SIGLEC5, sialic acid binding Ig‐like lectin 5; SNPs, single nucleotide polymorphisms; T2DM, Type 2 Diabetes Mellitus; TCF7L2, transcription factor 7‐like 2; TGM3, Transglutaminase 3; TRPS1, transcriptional repressor GATA binding 1.

### 3.2 Gingivitis

#### 3.2.1 ‘Diagnostic’ Studies (PICOTS#1)

Ten of the included studies investigated host genetic variants related to gingivitis. Eight of these studies investigated gingivitis in comparison with gingival health. Gingivitis and health definitions varied, as described in Table 2. Genetic variants investigated mainly included SNPs of cytokine genes such as *IL1*, *IL6* and *IL10*, as well as the *MMP* gene. Although some of the studies showed statistically significant associations between genotypes and the presence of gingivitis, no studies provided any data on diagnostic accuracy. Data could be extrapolated from contingency tables present in some of the papers reporting statistically significant associations. For example, considering *IL10* rs1800872 AA + AC (vs. CC) genotypes in Garlet et al., the accuracy for the diagnosis of gingivitis is 0.52 (0.49–0.56). When using *IL10* 1082 in the study by Dashash et al. (2005), GA + AA genotypes (vs. GG) resulted in an accuracy of 0.63 (95% CI: 0.57–0.69) with a sensitivity of 74.7% and specificity of 40.7% (Dashash et al. 2005; Garlet et al. 2012).

Two studies investigated the induction of experimental gingivitis (Jepsen et al. 2003; Scapoli et al. 2007) (Table 2). No statistically significant associations were detected between the investigated gene variants and development/clinical measures of experimental gingivitis.

### 3.3 Periodontitis

#### 3.3.1 Study Characteristics: ‘Diagnostic’ Studies (PICOTS#1)

Two papers (described in Table 3) specifically reported diagnostic genetic testing to assess the associations between host genetic variants and susceptibility to periodontitis (Li et al. 2020; Lopez et al. 2005) and provided diagnostic accuracy data. One study focused on a combination of genetic variants (Li et al. 2020), another investigated *IL1A* and *IL1B* polymorphisms in an adult Chilean Caucasian population consisting of 330 cases and 101 controls (Lopez et al. 2005), reporting a sensitivity of 26% and specificity of 90% for the diagnosis of periodontitis based on the genetic test (compared with the gold‐standard clinical definition), and accuracy calculated at 0.41 (0.36–0.46). The Li (2020) study investigated the association between the genetic risk score (GRS) and generalised aggressive periodontitis (GAgP) in an adult Chinese population, consisting of 335 GAgP patients and 114 healthy controls. The GRS was computed from four SNPs (matrix metalloproteinase 8 rs11225395, epidermal growth factor rs2237051, PPARa rs4253623 and apolipoprotein E rs429358), which were genotyped via the mass‐spectrometry‐based assay MassARRAY. In particular, the area under the curve (AUC) using the combined GRS was 0.62 (95% CI: 0.60–0.68), computed from the combination of sensitivity and specificity. Overall, it appeared that the GRS added very little to the AUC generated by other risk factors (age, sex, BMI, WBC, albulin/globulin, resulting in AUC of 0.69, increasing to 0.74 with the addition of the GRS) (Li et al. 2020).

An additional 597 papers were included based on this review's inclusion/exclusion criteria (Supplemental material 2 of Supporting Information). More detailed analysis was carried out in candidate gene studies including at least 1000 participants of specified ethnicity (*n* = 16), as reported in Table 3. Different sets of gene variants, often identified in previous GWAS, were analysed in the context of case–control studies (*n* = 6). Detailed information is given in Table 4. It was not possible to carry out meta‐analyses of these data across studies because of insufficient studies presenting data for the same genetic variants. However, diagnostic accuracy data could be derived from contingency tables in the papers. Overall, making assumptions about risk genotype in each analysis, sensitivity ranged between 0.10 and 0.90, specificity from 0.17 to 0.89 and accuracy from 0.25 to 0.65; no study had high values for both sensitivity and specificity. For example, using contingency tables from Tong and co‐workers for SIGLEC5 rs4284742 (GG genotype), the values for sensitivity, specificity and accuracy were 0.68, 0.38 and 0.49 (0.43–0.51), respectively (Tong et al. 2019). In the study by Schaefer et al. (2010) on DEFB1, the GG genotype for rs1047031 (vs. GA + AA) had sensitivity, specificity and accuracy of 0.63, 0.31 and 0.41 (0.40–0.43), respectively.

#### 3.3.2 Study Characteristics: ‘Progression Studies’ (PICOTS#2)

Sixteen studies presented data on the progression of periodontitis patients, either without any specific treatment provided or during supportive periodontal care following non‐surgical periodontal therapy and/or additional treatment (references are given in Table 5). Sample sizes varied from 20 to 1117 patients. Genetic variants investigated mainly included SNPs of cytokine genes. Although some of the studies showed statistically significant associations between genotypes and disease progression, no studies provided any data on diagnostic accuracy.

**TABLE 5:** Parameters of candidate gene studies included in the systematic review based on studies for PICOTS#2 and # 3 for periodontitis.

Authors, year of publication, PMID	Number of parcipitans, ethnicity	Study type	Gene variants	Time of follow‐up	Treatment	Measurements	Results
Walther et al. 2022 36101477	*n* = 209 Caucasians	Treatment response/progression	IL‐1, IL4, GATA‐3 and COX‐2 SNPs	27.5 months	NSPT + syst AB, then SPT	CAL, % sites with further attachment loss (PSAL) ≥ 1.3 mm, BOP and plaque score	GATA‐3 heterozygous genotypes and SNPs than wild‐type: lower CAL
Chatzopoulos et al. 2022 36101477	*n* = 37 CP Caucasians	Treatment response/progression	IL‐6 (rs1800796) and IL‐10 (rs1800872)	3 years	OHI + NSPT	PPD, CAL and BOP (patients divided into ‘at risk’ vs. ‘not at risk’)	No associations
Gorgun et al. 2021 34290170	*n* = 130 AgP *n* = 120 CP *n* = 70 H Turkish	Treatment response	IL‐13 (−1112 C/T and −1512 A/C)	6 weeks	NSPT	PI, GI, PPD, CAL	No associations
Wang et al. 2020 32077489	*n* = 125 GAgP Chinese	Treatment response/progression	CYP1A1 rs1048943	3 years (*n* = 81)	NSPT	BI, PPD, tooth loss	G allele carriers: higher BI, higher mean PPD in PD ≥ 7 mm at 3 years
Yoshihara et al. 2019 31002408	*n* = 294 Japanese	Treatment response/progression	β‐3 adrenergic receptor SNP	6 years	No specific treatment	Incidence rate ratio (IRR) for periodontal disease events	Dose–response relationship between β‐3 adrenergic receptor genotypes and periodontitis progression based on smoking years
Chatzopoulos et al. 2018 29624676	*n* = 67 CP Caucasians	Treatment response	IL‐6 (rs1800796) and IL‐10 (rs1800872)	45 days	NSPT	BOP, PPD, CAL	Carriers of the IL‐10‐592A allele: association with BOP ≥ 30%
Chang et al. 2018 28427824	*n* = 40 AgP‐CP Chinese	Treatment response	MCP‐1‐2518 SNP	6 weeks	NSPT	PPD, CAL, GI, BI, PI	Association of genotypes with treatment response in AgP (PD, GI, BI)
Emingil et al. 2014 24283658	*n* = 100 GAgP Turkish from Aegean region	Treatment response	MMP‐8 and MMP‐1 (TIMP‐1) SNPs	6 months	NSPT	PPD, CAL, BOP, PI	Associations of alleles with GCF MMP‐8 levels
Giannobile et al. 2013 23752171	*n* = 5.117 USA, 96% and 98% Caucasians, respectively	Progression	IL‐1 genotype (considered risk factors: genotype positive, smoking, diabetes mellitus)	16 years	No specific treatment	Tooth loss	Increasing tooth loss by increasing risk factors; bi‐annual dental check‐ups reduce the risk for tooth loss in genotype‐positive patients
Nibali et al. 2013 22918663	*n* = 12 UK Caucasians	Treatment response	IL6 haplotypes	160 days	NSPT + OFD	PPD, CAL, BOP	Association with CAL only at baseline
Corbi et al. 2014 24033189	*n* = 41 CP Brazilian	Treatment response	IL8 ATC/TTC haplotype	45 days	NSPT	PPD, CAL, GI, PI	No associations
Finoti et al. 2013 23821559	*n* = 30 Brazilian	Treatment response	IL8 ATC/TTC haplotype	45 days	NSPT	PPD, CAL, GI, PI	No associations
Ozkaca et al. 2010 20132924	*n* = 83 CP Turkish	Treatment response	MBL gene exon‐1 polymorphisms	6 months	NSPT	PPD, CAL, BOP	No associations
Nishida et al. 2010 20042735	*n* = 183 Japanese	Progression	ALDH2 genotypes	4 years	No specific treatment	Progression defined as ≥ 2 teeth with increase in PPD of ≥ 2 mm	No associations
Cullinan et al. 2007 18447856	*n* = 252 Australian, Caucasian	Treatment response/progression	IL10 −592 and −1082 SNPs	5 years	Oral Care Research Programme (unclear procedures)	PPD, CAL	Associations between IL10 haplotypes and PPD ≥ 4 mm
Eickholz et al. 2008 18199150	*n* = 100 (95 Caucasian)	Treatment response/progression	IL1 genotypes	10 years	SPC	PPD, BOP, tooth loss	Association between IL1 genotypes and tooth loss
Kratka et al. 2007 17575917	*n* = 20 with EOP (age 15–26 years)	Treatment response/progression	IL1A and B genotypes	10 years	Periodontal treatment and maintenance	PPD	Higher prevalence of composite IL1 genotype in EOP patients with progressive disease
Yoshie et al. 2007	*n* = 49 Japanese	Treatment response	IL‐1A +4845 G/T genotypes	4 weeks	SRP	PPD, CAL, BOP	No associations
Wolf et al. 2006	*n* = 132 CP Northern European Caucasians	Treatment response/progression	SNPs in Fcgamma receptor IIa (131R/H) and IIIb (NA1/NA2)	4 and 30 months	OHI, extractions, SRP, systemic AB and surgery when needed	PPD, CAL	No associations
Persson et al. 2003	224	Progression	IL‐1 genotypes	4 years	SPT	Periodontal risk assessment (PRA, incl. age related bone level and gender), PPD, BOP	IL‐1 genotype no influence on PPD and BOP, but negatively influenced PRA
Inagaki et al. 2003	*n* = 125 Japanese men	Progression	VDR (ApaI, TaqI)	23 years	Oral examinations	PPD, CAL, BOP, ABL	VDR (ApaI) associated with bone, attachment and tooth loss
Cullinan et al. 2001	*n* = 295 European heritage	Progression	IL‐1A +4845 IL‐1B +3954	5 years	Periodontal maintenance	PPD, CAL	No associations
Cattabriga et al. 2001	*n* = 60 Italian Caucasian	Progression	IL‐1 genotypes	10 years	NSPT, 36 surgical therapy	Radiographic examination (bone defect level, bone crest level)	No associations
Lang et al. 2000	*n* = 323 (subset of 139 never smokers)	Progression	IL‐1A (+4845) IL‐1B (+3954)	SPT	NSPT	PPD, CAL, BOP	No associations (subset analysis: increased BOP with positive genotype)
Ehmke et al. 1999	*n* = 48 German Caucasians	Treatment response/progression	IL‐1A gene (−889) IL‐1B gene (+3953)	2 years	NSPT: randomly assigned to test (Amoxi + Met + CHX) or control	PPD, CAL	No associations

Abbreviations: AB, antibiotic; ABL, alveolar bone loss; BOP, bleeding on probing; CAL, clinical attachment loss; CHX, chlorhexidine; GAgP, generalised aggressive periodontitis; GI, gingival index; NSPT, non‐surgical periodontal therapy; OFD, open flap debridement; PD, periodontitis; PI, plaque index; PPD, probing pocket depth; PRA, patient risk assessment.

#### 3.3.3 Study Characteristics: ‘Treatment Success’ (PICOTS#3)

Eighteen studies presented data on treatment response, including studies overlapping with those reported above as ‘progression studies’ (references are given in Table 4). Sample sizes varied from 12 to 323 patients. Genetic variants investigated mainly included SNPs of cytokine genes. Treatment usually consisted of oral hygiene instructions and non‐surgical periodontal therapy, plus in some cases systemic antibiotics and/or periodontal surgeries. One pilot study used genetic variants for study inclusion, ensuring a balanced number of IL6 haplotype positive and haplotype negative (Nibali et al. 2013) based on IL6 haplotypes. Although some of the studies showed statistically significant associations between genotypes and the presence of gingivitis, no studies provided any data on the predictive ability of genetic variants on disease progression. Data could be extrapolated from contingency tables present in some of the papers reporting statistically significant associations. For example, considering *ALDH2* 2/2 versus 1/1 + 1/2 genotypes (Nishida et al. 2010), accuracy for the diagnosis of gingivitis is 0.52 (0.45–0.60), with a sensitivity of 0.26 and a specificity of 0.89.

#### 3.3.4 Risk of Bias

For gingivitis, RoB analyses showed scores ranging from 15 to 19 on a scale up to 20 (Nibali 2013). A higher risk of bias was found mainly regarding power calculation, success rate of DNA extraction and genotyping blind to case–control status (Table 6).

**TABLE 6:** Results of RoB analysis (using Nibali 2013 scale for candidate gene studies) for studies analysed for gingivitis.

Study	PMID	Item and score										
		Adequate case definition	Representativeness of cases	Selection of controls	Definition of controls	Confounders	Ascertainment of exposure	Same method of ascertainment for cases and controls	Non‐response rate	Power calculation	Statistics	Corrected statistics
Becerra_Ruiz 2021	PMID: 34027804	1	1	1	1	1	1	1	0	0	1	0
Barbosa 2020	PMID: 32902920	1	1	1	1	1	1	1	0	1	1	1
Reis 2021	PMID: 32643487	1	1	1	1	1	1	1	0	1	1	0
Garlet 2012	PMID: 22324464	1	1	1	1	1	1	1	0	1	1	1
Vokurka 2009	PMID: 18930181	1	1	1	1	1	1	1	0	1	1	1
Izakovicova Holla 2008	PMID: 18446552	1	1	1	1	1	1	1	0	1	1	1
Dashash 2005	PMID: 16171432	1	1	1	1	1	1	1	0	0	1	1
Müller 2007	PMID: 17492470	1	1	1	1	1	1	1	0	0	1	1
Jepsen 2003	PMID: 12622850	1	1	1	1	1	1	1	0	0	1	n.e.
Scapoli 2007	PMID: 17953695	1	1	1	1	1	1	1	0	0	1	1

Study	Item and score										
	Odds ratios and confidence intervals	Success rate of DNA extraction	Success rate of genetic assessment	Genotype counts	Hardy–Weinberg equilibrium	Primer sequence	Reproducibility	Genotyping blind to case–control status	Proposed inheritance model	Total	Corrected total
Becerra_Ruiz 2021	1	0	0	1	1	1	1	1	1	15	15
Barbosa 2020	1	1	0	1	0	1	1	0	1	16	16
Reis 2021	1	0	0	1	1	1	1	0	1	15	15
Garlet 2012	1	1	1	1	1	1	1	1	1	19	19
Vokurka 2009	1	0	0	1	1	1	1	0	1	16	16
Izakovicova Holla 2008	1	1	1	1	1	1	1	0	1	18	18
Dashash 2005	1	0	0	1	1	1	1	0	1	15	15
Müller 2007	1	0	0	1	1	1	1	n.e.	1	15	16
Jepsen 2003	1	0	0	1	n.e.	1	1	n.e.	n.e.	12	16
Scapoli 2007	1	1	1	1	1	1	1	0	1	17	17

Abbreviation: n.e., not equickable.

For periodontitis (PICOTS#1), Table 7 reports RoB analyses, revealing scores ranging from 11 to 18 on a scale up to 20 (Nibali 2013). Most studies (*n* = 26) were scored between 11 and 14, while a smaller proportion (*n* = 18) scored between 15 and 18. The main aspects with potential RoB were lack of power calculation, corrected statistics, success rate of DNA extraction and of genetic assessment and genotyping blind to case–control status. It is also important to highlight that those definitions of periodontitis and health varied across the included studies (Li et al. 2020; Lopez et al. 2005; Nibali et al. 2013). Furthermore, some studies included smokers (Lopez et al. 2005), while other studies excluded smokers.

**TABLE 7:** Results of RoB analysis (using Nibali 2013 scale for candidate gene studies) for studies analysed for PICOTS#1 for periodontitis.

Study	PMID	Item and score										
		Adequate case definition	Represen‐tativeness of cases	Selection of controls	Definition of controls	Confounders	Ascertainment of exposure	Same method of ascertainment for cases and controls	Non‐response rate	Power calculation	Statistics	Corrected statistics
Asa'ad 2023	PMID: 37382343	1	1	1	1	1	1	1	1	0	1	1
Yang 2022	PMID: 35179257	1	1	1	1	0	1	1	1	0	1	1
She 2021	PMID: 33854406	1	1	1	0	1	1	1	0	1	1	0
Tong 2019	PMID: 30765789	1	1	1	1	1	1	1	1	0	1	1
Folwaczny 2018	PMID: 28983640	1	1	1	0	1	1	1	0	1	1	1
Schulz 2018	PMID: 2973256	1	1	1	0	1	1	1	1	0	1	1
Tanaka 2017	PMID: 29129846	0	1	1	1	1	1	1	0	1	1	1
Shang 2015	PMID: 26643602	1	1	1	0	1	1	1	0	1	1	1
Wu 2015	PMID: 24690098	1	1	1	1	1	1	1	0	0	1	1
Tanaka 2014	PMID: 24460370	0	1	1	0	1	1	1	0	1	1	1
Zhang 2014	PMID: 25101955	1	1	1	1	1	1	1	0	1	1	1
Folwaczny 2011a	PMID: 21954916	1	1	1	1	1	1	1	1	1	1	1
Folwaczny 2011b	PMID: 21388352	1	1	1	1	1	1	1	0	1	1	1
Folwaczny 2011c	PMID: 21252350	1	1	0	1	1	1	1	0	1	1	1
Schaefer 2010	PMID: 19829306	1	1	1	1	1	1	1	0	1	1	1
Schaefer 2009	PMID: 19214202	1	1	1	1	1	1	1	0	1	1	1

Study	Item and score										
	Odds ratios and confidence intervals	Success rate of DNA extraction	Success rate of genetic assessment	Genotype counts	Hardy–Weinberg equilibrium	Primer sequence	Reproducibility	Genotyping blind to case–control status	Proposed inheritance model	Total	Corrected total
Asa'ad 2023	1	0	0	1	1	1	1	0	1	16	16
Yang 2022	1	1	1	n.e.*	1	n.e.*	1	1	n.e.*	14	17
She 2021	1	1	1	1	1	1	1	1	1	17	17
Tong 2019	1	0	0	1	1	1	1	1	1	16	16
Folwaczny 2018	1	0	0	1	1	1	1	0	1	15	15
Schulz 2018	0.5	0	0	1	1	1	1	0	1	14.5	14.5
Tanaka 2017	1	0	0	1	1	1	1	0	1	15	15
Shang 2015	0.5	0	0	1	1	1	1	1	1	15.5	15.5
Wu 2015	1	0	1	1	1	1	1	1	1	17	17
Tanaka 2014	1	0	0	1	1	1	1	0	1	14	14
Zhang 2014	1	1	1	1	1	1	1	1	1	19	19
Folwaczny 2011a	1	0	1	1	1	1	1	0	1	18	18
Folwaczny 2011b	1	0	0	1	1	1	1	0	0	15	15
Folwaczny 2011c	1	0	0	1	1	0	1	0	1	14	14
Schaefer 2010	1	0	0	1	1	1	1	0	1	16	16
Schaefer 2009	1	0	0	1	1	1	1	0	1	16	16

Abbreviations: n.e.; not assessed, because the study type of Yang et al. 2022 is GWAS.

For periodontitis (PICOTS#2 and #3), RoB analyses showed scores ranging from 5 to 14 on a scale up to 20 (Nibali 2013). A lack of reported information was identified for 9 out of 20 items related to candidate gene studies (Table 8).

**TABLE 8:** Results of RoB analysis (using Nibali 2013 scale for candidate gene studies) for studies analysed for PICOTS#2 and #3 for periodontitis.

Study	PMID	Item and score										
		Adequate case definition	Represen‐tativeness of cases	Selection of controls	Definition of controls	Confounders	Ascertainment of exposure	Same method of ascertainment for cases and controls	Non‐response rate	Power calculation	Statistics	Corrected statistics
Walther 2022	PMID: 36101477	1	1	1	1	1	1	1	0	1	1	1
Chatzopoulos 2022	PMID: 35686958	1	1	0	0	1	1	1	0	0	1	0
Gorgun 2021	PMID: 34290170	1	1	1	1	1	1	1	0	1	1	0
Wang 2020	PMID: 32077489	1	1	1	1	1	1	1	0	0	1	1
Yoshihara 2019	PMID: 31002408	1	1	0	0	1	1	1	1	0	1	1
Chatzopoulos 2018	PMID: 29624676	1	1	0	0	1	1	1	0	1	1	1
Chang 2018	PMID: 28427824	1	1	0	0	1	1	1	0	1	1	1
Emingil 2014	PMID: 24283658	1	1	1	1	1	1	1	1	1	1	1
Giannobile 2013	PMID: 23752171	1	1	1	1	1	1	1	1	0	1	1
Nibali 2013	PMID: 22918663	1	1	1	1	1	1	1	1	1	1	1
Corbi 2014	PMID: 24033189	1	1	1	1	1	1	1	1	1	1	1
Finoti 2013	PMID: 23821559	1	1	0	0	1	1	1	1	0	1	0
Ozkaca 2010	PMID: 20132924	1	1	1	1	1	1	1	1	0	1	1
Nishida 2010	PMID: 20042735	1	1	0	0	1	1	1	1	0	1	1
Cullinan 2007	PMID: 18447856	1	1	0	0	1	1	1	1	0	1	1
Eickholz 2008	PMID: 18199150	1	1	0	0	1	1	1	1	0	1	1
Kratka 2007	PMID: 17575917	1	1	0	0	0	1	1	0	0	1	0
Yoshie 2007	PMID: 17335373	1	1	0	0	1	1	1	1	0	1	1
Wolf 2006	PMID: 16889631	1	1	1	1	1	1	1	1	0	1	1
Persson 2003	PMID: 15643745	1	1	0	0	1	1	1	0	0	1	0
Inagaki et al. 2003	PMID: 12666703	1	1	1	1	1	1	1	1	0	1	1
Cullinan et al. 2001	PMID: 11737511	1	1	0	0	1	1	1	1	0	1	0
Cattabriga et al. 2001	PMID: 11453239	1	1	0	0	1	1	1	0	0	1	1
Lang et al. 2000	PMID: 10863964	1	1	0	0	1	1	1	1	0	1	1
Ehmke et al. 1999	PMID: 10599909	1	1	0	0	0	1	1	0	0	0	0

Study	Item and score									
	Odds ratios and confidence intervals	Success rate of DNA extraction	Success rate of genetic assessment	Genotype counts	Hardy–Weinberg equilibrium	Primer sequence	Reproducibility	Genotyping blind to case–control status	Proposed inheritance model	Total
Walther 2022	0	0	0	1	0	0	1	1	0	13
Chatzopoulos 2022	0	0	0	1	0	0	1	0	0	8
Gorgun 2021	0	0	0	1	1	1	1	0	1	14
Wang 2020	0	0	0	0	0	0	0	0	0	9
Yoshihara 2019	0	0	1	1	0	0	1	0	0	11
Chatzopoulos 2018	1	0	0	1	0	1	1	0	0	12
Chang 2018	0	0	0	1	0	1	0	0	0	10
Emingil 2014	1	0	0	1	0	0	1	0	0	14
Giannobile 2013	0	0	0	0	0	0	1	0	0	11
Nibali 2013	0	0	0	1	0	0	1	1	0	14
Corbi 2014	1	0	0	1	0	0	0	0	0	13
Finoti 2013	0	0	0	1	0	0	0	0	0	8
Ozkaca 2010	1	0	0	1	0	1	1	0	0	14
Nishida 2010	1	0	0	1	0	0	1	0	0	11
Cullinan 2007	0	0	0	1	0	0	1	0	0	10
Eickholz 2008	1	0	0	0	0	0	0	0	0	9
Kratka 2007	0	0	0	0	0	0	0	0	0	5
Yoshie 2007	0	0	0	1	0	0	1	0	0	10
Wolf 2006	0	0	0	1	0	1	1	1	0	14
Persson 2003	0	0	0	0	0	0	0	0	0	6
Inagaki et al. 2003	0	0	0	1	0	1	1	0	0	13
Cullinan et al. 2001	0	0	0	1	0	0	1	0	0	9
Cattabriga et al. 2001	0	0	0	1	0	0	0	1	0	9
Lang et al. 2000	0	0	0	0	0	1	1	1	0	11
Ehmke et al. 1999	0	0	0	1	0	0	0	0	0	5

Table 9 reports the RoB analysis using the QADAS‐2 score for the two studies identified as diagnostic/prognostic, showing a low overall RoB.

**TABLE 9:** Risk‐of‐bias analysis using QADAS‐2 score for the two studies identified as diagnostic/prognostic.

Domains	1. Patient selection		2. Index test		3. Reference standard		
	Risk of bias	Applicability	Risk of bias	Applicability	Risk of bias	Applicability	4. Flow and timing
Li, 2020	Unclear	Low	Low	Low	Low	Low	Low
Lopez, 2005	Unclear	Low	Low	Low	Low	Low	Low

## 4 Discussion

We are at a crucial crossroads in health care and in dentistry, where omics technologies are providing unique opportunities to shift management from empirical one‐size‐fits‐all approaches to personalised treatment plans. This review aimed to investigate whether, at this important junction, genetic tests could help in the diagnosis and management of periodontal diseases. This is of special interest, given that approximately a third of the variance for the risk of periodontitis in the population (and a similar estimate for gingivitis too, albeit with weaker evidence) is attributable to host genetic factors (Nibali et al. 2019).

Genetic diagnosis is already a reality for very rare syndromic or non‐syndromic polygenic forms of periodontitis (Schaefer et al. 2025). However, this systematic review shows that considerable advancements and investments are needed to move this research field forward. It must be stressed that, in the great majority of periodontitis cases, genomic information is best suited for providing estimates of susceptibility for the onset, severity, progression and treatment response for the common forms of periodontal diseases, rather than diagnosis; for the latter, biomarkers that are proximal to disease development or actually reflective of disease presence would be better suited.

From an extensive, systematic search of the literature, only two papers were identified that provided data on genetic ‘diagnostic’ accuracy in cases of periodontal disease, both focusing on periodontitis. One examined a single host genetic variant (*IL1* gene) (Lopez et al. 2005) and another a polygenic score (Li et al. 2020). None of these included chair‐side tests. ‘Diagnostic’ ability in these studies was measured by sensitivity/specificity and positive/negative predictive values in one study (Lopez et al. 2005) and by AUC in the other (Li et al. 2020). Lopez et al. (2005) showed a high specificity, meaning that a ‘negative’ genetic test result usually corresponded with a ‘negative’ history of periodontitis, but a low sensitivity, meaning that a ‘positive’ genetic test often did not correspond with the periodontitis diagnosis. In the study by Li et al. (2020), the AUC for detection of periodontitis by the genetic test was not better than acceptable according to suggested standards (Hosmer et al. 2013). The true biological effects of the genetic variants tested in the two identified publications remain unclear, or may not be relevant, as none of those has been found to be of genome‐wide significance in GWAS so far.

Because of the paucity of ‘diagnostic studies’ as such, we extracted diagnostic/susceptibility data from studies reporting genotype frequency in cases and controls. According to the characteristics of study design, this was done in hypothesis‐testing candidate gene studies, rather than in GWAS, which are hypothesis‐free, unbiased and cover the entire genome. By contrast, candidate gene studies have their limitations, namely potentially hypothesising incorrect portions of the genome and incorrect effect sizes, as well as being affected by ‘winners curse’ and reporting bias. Further, the candidate gene studies lack the methodological rigour required to allow for small differences in ancestry and relatedness, which can lead to confounding (Duncan et al. 2019).

An attempt to derive summary measures for sensitivity and specificity was carried out by selecting, out of the 561 originally identified papers, 16 that had a sample size of at least 1000 individuals, thus having increased power. Although the definition of ‘true positives’ and ‘false negatives’ lends itself to interpretation in this context (wild/rare allele homozygosity, allele frequency), results were generally underwhelming, with low to moderate sensitivity and specificity in genotypes or haplotypes. Also, when the potential value of gene variants identified in GWAS and then replicated in CGS and identified in an additional search was assessed from a diagnostic point of view, their diagnostic value (sensitivity, specificity) was still found to be limited (Tong et al. 2019). Therefore, even though gene loci identified in published GWAS as associated with periodontitis‐related phenotypes are replicated successfully in independent populations (Yang et al. 2022), their diagnostic value may still be limited. This limited diagnostic value could be based on the approach to test single genetic variations in relation to periodontitis, which is considered a complex inflammatory disease. Periodontitis is clearly a polygenic disease with a substantial behavioural/environmental component. Therefore, diagnostic accuracy may be increased only when considering composite genotypes.

It is conceivable that diagnostic genetic tests could be developed for severe, single‐gene, early onset forms of periodontitis in patients with a large genetic burden. For example, a number of papers consistently reported on a variant in the *CTSC* gene in association with non‐syndromic early onset periodontitis (Hewitt et al. 2004; Molitor et al. 2019; Noack et al. 2008; Richter et al. 2022). Those rare mutation variants possess great potential to serve as additional modifying factors for periodontitis grading (Tonetti et al. 2018). A promising diagnostic genetic test for people who have a very high risk of developing periodontitis in adolescence or as young adults could be developed for mutations in the *CTSC* gene and potentially other mutations leading to early onset forms of periodontitis (Schäfer et al. 2025). However, to date, no studies providing data for this specific scenario are available in the literature.

Only one pilot study selected patients based on their genetic profile (dividing them into ‘*IL6* positive’ and ‘*IL6* negative’ based on *IL6* haplotypes) and assessed their response to periodontal treatment, showing a difference between groups for clinical and inflammatory data post non‐surgical and surgical therapy (Nibali et al. 2019). Among other prognostic studies, a much larger retrospective study showed that high‐risk patients (as identified by a combination of smoking, diabetes and IL1 genotypes) benefitted more from two recall visits per year compared with low‐risk patients (Giannobile et al. 2013). However, the benefit of adding the genetic parameter (IL1 genotypes) to other susceptibility factors appeared to be limited (Diehl et al. 2015).

Polygenic risk scores (PRSs) may allow for stratification of individuals with different onset and trajectories of periodontitis with great potential for genetic screening in early life to indicate risk prediction (Inouye et al. 2018; Khera et al. 2018). A lesson can be learned from other polygenic diseases, integrating the risk conveyed by a large number of genetic variants, for example, those genotyped by GWAS, into a single variable (International Schizophrenia et al. 2009; Wray et al. 2007). Ideally, PRSs can provide a simple but comprehensive assessment of the genetic risk of the individual, serving as a proxy for the genetic load of a trait at the individual level. Specifically, PRSs show predictive value for numerous common diseases (Lambert et al. 2019). In the Polygenic Score (PGS) catalogue (https://www.pgscatalog.org) (Lambert et al. 2021), there are currently a total of 4857 polygenic (risk) scores for 655 diseases and traits (as of September 2024). To date, no PRS for periodontitis has been developed. However, a recent study showed how the addition of predisposing genetic loci in a model of periodontitis susceptibility can increase the AUC and thus the predictive ability of the model (Morelli et al. 2020). This led to increases in AUC of > 0.9 with the inclusion of 100 loci. However, these data have not been validated or externally replicated in independent populations.

Evidently, the robustness of a PRS depends directly on the quality of the genetic data on which it is based, and its implementation in clinical settings and healthcare systems is still not routine (Lambert et al. 2019; Ritchie et al. 2024; Roberts et al. 2023). PRSs are not suitable for diagnosing a disease but can only predict a person's genetic likelihood of developing a disease over time, which is further influenced by non‐genetic factors. Genome‐wide PRSs are discussed to improve polygenic risk prediction to facilitate clinical care. Large GWAS with > 100,000 participants hold the basis for the identification and validation of risk variants of common diseases with a strong environmental component, such as coronary artery disease (CAD), atrial fibrillation and type 2 diabetes mellitus (T2DM) (Khera et al. 2018). For coronary heart disease (CHD), genetic risk prediction was carried out by applying GRS including 27, 28 or 50 genetic variants (Tada et al. 2016; Tikkanen et al. 2013). In these studies, 23,595 or 24,124 participants, respectively, were evaluated during follow‐up periods of up to 14 years. During follow‐up, CHD events, such as acute coronary syndrome, were observed, and genetic information was adjusted for additional risk factors as well as family history for CHD. This approach allowed for early risk prediction and identification of individuals at high risk for the first CHD event (Tada et al. 2016; Tikkanen et al. 2013). Improved performance for PRS has more recently been shown using genome‐wide prediction methods and matrices and using genetically correlated traits (Albinana et al. 2023; Zabad et al. 2023).

When applying a similar approach for a PRS for periodontitis, it would be important not only to analyse cross‐sectional data but also to include prospective information. Like CHD, periodontitis is an age‐related (but not dependent) disease that is linked to additional risk factors such as smoking and diabetes mellitus (Graziani et al. 2018; Palmer et al. 2005). This aspect is of importance when considering early onset forms of rapidly progressing (grade C) periodontitis. In cross‐sectional studies, the comparison of young patients with grade C periodontitis with healthy individuals of the same age will not allow for excluding the possibility of periodontitis occurring at a later stage in life. Thus, studies that cover long time periods are needed to reveal relationships between genetic risk variants, the accumulation of risk factors during life and the first onset of inflammatory periodontal destruction.

Currently, the available dataset for GWAS related to early onset (grade C) periodontitis identified potentially predisposing variants in the genes of DEFA1A3, FCER1G and SIGLEC5 at the level of genome‐wide significance. However, the number of individuals included is relatively low when compared to GWAS for the above‐mentioned diseases (De Almeida et al. 2024; Munz et al. 2018; Shungin et al. 2019). It is conceivable that with increasing power, the number of genetic variants that reach genome‐wide significance will be higher for periodontitis (Munz et al. 2017; Schaefer et al. 2010). In the context of periodontal disease onset, the true biological effects of the identified genetic variants are yet to be explored. The known biological functions of the identified genes direct to important innate and adaptive immune responses, which allow for speculations regarding the biological effects of the genetic variants (Crocker et al. 2007; De Almeida et al. 2024; Junker et al. 2020; Munz et al. 2018; Nemeth et al. 2019; Tong et al. 2019; Vuchkovska et al. 2022). It is clear that one, two, or even all three identified genetic variants alone will not provide high genetic effects and thus will just predispose rather than cause the periodontitis phenotype. In conjunction with other risk factors, future studies with greater power may reveal more genetic variants, bring those into a clinical context and known risk factors for periodontitis and eventually help create genetic risk scores for periodontitis.

The findings of this review should be interpreted against the backdrop of the risk of bias and methodological flaws of included papers, mainly related to potential positive publication bias, small sample sizes and lack of power calculation, lack of replication samples and details about statistics and genotyping. Furthermore, none of the available assessment tools fulfils each criterion necessary to evaluate the qualitative nature of genetic tests (see Box 1).

A total of 10 publications on genetic analyses in gingivitis were included. Eight of these studies investigated gingivitis in comparison with gingival health, and the genetic variants investigated mainly included SNPs of cytokine genes, such as *IL1*, *IL6* and *IL10*, as well as an *MMP* gene. No study reported any data on diagnostic accuracy. Extrapolated data allowed the calculation of data on diagnostic accuracy by the reviewers, and it was found that the accuracy of diagnosis showed wide variability. As an example, in the study by Dashash et al. (2005) on *IL10* 1082, the GA + AA genotypes (vs. GG) resulted in an accuracy of 0.63 (95% CI: 0.57–0.69) with sensitivity 74.7% and specificity 40.7%. The two identified studies on the induction of experimental gingivitis (Jepsen et al. 2003; Scapoli et al. 2007) did not show any association between the investigated gene variants and development/clinical measures of experimental gingivitis. Genetic studies on the transformation from health to gingivitis, as well as the distinction between gingivitis and periodontitis, remain an important area for future research to reflect the suspected importance of host genetic variants in its susceptibility (Nibali et al. 2019).

The strength of this review is the novel approach attempting to assess the diagnostic value of genetic testing for periodontal disease. Limitations pertain to the lack of strictly ‘diagnostic’ studies in this field and the high heterogeneity and risk of bias among the included studies. With the applied search strategy, only two articles were found that reported an assessment of diagnostic accuracy, while in several other studies values for diagnostic accuracy were calculated by the reviewers, which might have introduced a potential risk of bias due to the complexity of phenotypes, ethnicity, power and genetic variants within those studies.

## 5 Conclusion

The main conclusions from this review are the following:

- No genetic tests are currently available that can reliably distinguish between different forms of periodontal disease and health (focused question 1), discriminate between disease progression or stability (focused question 2) or be used for the prediction of disease resolution (focused question 3);
- Identification of rare variants in families with known risk for early onset periodontitis may be useful for individual diagnostic and preventive measures because of the strong effect sizes;
- Promising gene variants identified in GWAS may form the basis for polygenic risk scores as a valid future diagnostic/prognostic options for periodontitis.

## Author Contributions

L.N. and H.D. contributed to conception of the SR protocol and study design, title and abstract search, full text reading, data analysis and interpretation and conception and writing the manuscript. D.H. contributed to literature search, title and abstract search, full text reading, data extraction, statistical analysis and interpretation. J.K. contributed to risk‐of‐bias analyses, statistical analysis and interpretation. E.M.‐C.L. contributed to literature search, title and abstract search, full text reading, risk‐of‐bias analyses and data extraction. A.S. and G.R. contributed to genetic data analysis and interpretation. All authors critically reviewed the manuscript.

## Conflicts of Interest

The authors declare no conflicts of interest.

## Supporting information


Data S1.

## Data Availability

Data sharing not applicable to this article as no datasets were generated or analysed during the current study.

## References

[jcpe14149-bib-0001] Albinana, C. , Z. Zhu , A. J. Schork , et al. 2023. “Multi‐PGS Enhances Polygenic Prediction by Combining 937 Polygenic Scores.” Nature Communications 14, no. 1: 4702. 10.1038/s41467-023-40330-w. PMC10404269 37543680

[jcpe14149-bib-0002] Arias‐Bujanda, N. , A. Regueira‐Iglesias , C. Balsa‐Castro , L. Nibali , N. Donos , and I. Tomas . 2019. “Accuracy of Single Molecular Biomarkers in Gingival Crevicular Fluid for the Diagnosis of Periodontitis: A Systematic Review and Meta‐Analysis.” Journal of Clinical Periodontology 46, no. 12: 1166–1182. 10.1111/jcpe.13188. 31444912

[jcpe14149-bib-0060] Aronson, J. K. , and R. E. Ferner . 2017. “Biomarkers‐A General Review.” Current Protocols in Pharmacology 76: 9.23.1–9.23.17. 10.1002/cpph.19. 28306150

[jcpe14149-bib-0003] Buniello, A. , J. A. L. MacArthur , M. Cerezo , et al. 2019. “The NHGRI‐EBI GWAS Catalog of Published Genome‐Wide Association Studies, Targeted Arrays and Summary Statistics 2019.” Nucleic Acids Research 47, no. D1: D1005–D1012. 10.1093/nar/gky1120. 30445434 PMC6323933

[jcpe14149-bib-0004] Crocker, P. R. , J. C. Paulson , and A. Varki . 2007. “Siglecs and Their Roles in the Immune System.” Nature Reviews. Immunology 7, no. 4: 255–266. 10.1038/nri2056. 17380156

[jcpe14149-bib-0005] Dashash, M. , A. S. Blinkhorn , I. V. Hutchinson , V. Pravica , and D. B. Drucker . 2005. “The Relationship Between Interleukin‐10 Gene Polymorphism at Position −1082 and Susceptibility to Gingivitis in Children.” Journal of Periodontology 76, no. 9: 1455–1462. 10.1902/jop.2005.76.9.1455. 16171432

[jcpe14149-bib-0006] De Almeida, S. D. , G. M. Richter , A. de Coo , et al. 2024. “A Genome‐Wide Association Study Meta‐Analysis in a European Sample of Stage III/IV Grade C Periodontitis Patients </=35 Years of Age Identifies New Risk Loci.” Journal of Clinical Periodontology 51, no. 4: 431–440. 10.1111/jcpe.13922. 38140892

[jcpe14149-bib-0007] Diehl, S. R. , F. Kuo , and T. C. Hart . 2015. “Interleukin 1 Genetic Tests Provide no Support for Reduction of Preventive Dental Care.” Journal of the American Dental Association (1939) 146, no. 3: 164–173. 10.1016/j.adaj.2014.12.018. 25726343

[jcpe14149-bib-0008] Duncan, L. E. , M. Ostacher , and J. Ballon . 2019. “How Genome‐Wide Association Studies (GWAS) Made Traditional Candidate Gene Studies Obsolete.” Neuropsychopharmacology 44, no. 9: 1518–1523. 10.1038/s41386-019-0389-5. 30982060 PMC6785091

[jcpe14149-bib-0009] Fuchsberger, C. , J. Flannick , T. M. Teslovich , et al. 2016. “The Genetic Architecture of Type 2 Diabetes.” Nature 536, no. 7614: 41–47. 10.1038/nature18642. 27398621 PMC5034897

[jcpe14149-bib-0010] Gao, C. , M. Iles , H. Larvin , et al. 2024. “Genome‐Wide Association Studies on Periodontitis: A Systematic Review.” PLoS One 19, no. 9: e0306983. 10.1371/journal.pone.0306983. 39240858 PMC11379206

[jcpe14149-bib-0011] Garlet, G. P. , A. P. Trombone , R. Menezes , et al. 2012. “The Use of Chronic Gingivitis as Reference Status Increases the Power and Odds of Periodontitis Genetic Studies – A Proposal Based in the Exposure Concept and Clearer Resistance and Susceptibility Phenotypes Definition.” Journal of Clinical Periodontology 39, no. 4: 323–332. 10.1111/j.1600-051X.2012.01859.x. 22324464

[jcpe14149-bib-0012] Genco, R. J. , and W. S. Borgnakke . 2013. “Risk Factors for Periodontal Disease.” Periodontology 2000 62, no. 1: 59–94. 10.1111/j.1600-0757.2012.00457.x. 23574464

[jcpe14149-bib-0013] Genovese, G. , M. Fromer , E. A. Stahl , et al. 2016. “Increased Burden of Ultra‐Rare Protein‐Altering Variants Among 4,877 Individuals With Schizophrenia.” Nature Neuroscience 19, no. 11: 1433–1441. 10.1038/nn.4402. 27694994 PMC5104192

[jcpe14149-bib-0014] Giannobile, W. V. , T. M. Braun , A. K. Caplis , L. Doucette‐Stamm , G. W. Duff , and K. S. Kornman . 2013. “Patient Stratification for Preventive Care in Dentistry.” Journal of Dental Research 92, no. 8: 694–701. 10.1177/0022034513492336. 23752171 PMC3711568

[jcpe14149-bib-0015] Graziani, F. , S. Gennai , A. Solini , and M. Petrini . 2018. “A Systematic Review and Meta‐Analysis of Epidemiologic Observational Evidence on the Effect of Periodontitis on Diabetes an Update of the EFP‐AAP Review.” Journal of Clinical Periodontology 45, no. 2: 167–187. 10.1111/jcpe.12837. 29277926

[jcpe14149-bib-0016] Hewitt, C. , D. McCormick , G. Linden , et al. 2004. “The Role of Cathepsin C in Papillon‐Lefèvre Syndrome, Prepubertal Periodontitis, and Aggressive Periodontitis: Mutations of Cathepsin C in Periodontitis.” Human Mutation 23, no. 3: 222–228. 10.1002/humu.10314. 14974080

[jcpe14149-bib-0017] Hosmer, D. W., Jr. , S. Lemeshow , and R. X. Sturdivant . 2013. Applied Logistic Regression, 1–510. John Wiley & Sons, Inc.

[jcpe14149-bib-0018] Inouye, M. , G. Abraham , C. P. Nelson , et al. 2018. “Genomic Risk Prediction of Coronary Artery Disease in 480,000 Adults: Implications for Primary Prevention.” Journal of the American College of Cardiology 72, no. 16: 1883–1893. 10.1016/j.jacc.2018.07.079. 30309464 PMC6176870

[jcpe14149-bib-0019] International Schizophrenia, C , S. M. Purcell , N. R. Wray , et al. 2009. “Common Polygenic Variation Contributes to Risk of Schizophrenia and Bipolar Disorder.” Nature 460, no. 7256: 748–752. 10.1038/nature08185. 19571811 PMC3912837

[jcpe14149-bib-0020] Jepsen, S. , J. Eberhard , D. Fricke , J. Hedderich , R. Siebert , and Y. Acil . 2003. “Interleukin‐1 Gene Polymorphisms and Experimental Gingivitis.” Journal of Clinical Periodontology 30, no. 2: 102–106. 10.1034/j.1600-051x.2003.00218.x. 12622850

[jcpe14149-bib-0021] Junker, F. , J. Gordon , and O. Qureshi . 2020. “Fc Gamma Receptors and Their Role in Antigen Uptake, Presentation, and T Cell Activation.” Frontiers in Immunology 11: 1393. 10.3389/fimmu.2020.01393. 32719679 PMC7350606

[jcpe14149-bib-0022] Khera, A. V. , M. Chaffin , K. G. Aragam , et al. 2018. “Genome‐Wide Polygenic Scores for Common Diseases Identify Individuals With Risk Equivalent to Monogenic Mutations.” Nature Genetics 50, no. 9: 1219–1224. 10.1038/s41588-018-0183-z. 30104762 PMC6128408

[jcpe14149-bib-0023] Lambert, S. A. , G. Abraham , and M. Inouye . 2019. “Towards Clinical Utility of Polygenic Risk Scores.” Human Molecular Genetics 28, no. R2: R133–R142. 10.1093/hmg/ddz187. 31363735

[jcpe14149-bib-0024] Lambert, S. A. , L. Gil , S. Jupp , et al. 2021. “The Polygenic Score Catalog as an Open Database for Reproducibility and Systematic Evaluation.” Nature Genetics 53, no. 4: 420–425. 10.1038/s41588-021-00783-5. 33692568 PMC11165303

[jcpe14149-bib-0025] Li, W. , X. Wang , Y. Tian , et al. 2020. “A Novel Multi‐Locus Genetic Risk Score Identifies Patients With Higher Risk of Generalized Aggressive Periodontitis.” Journal of Periodontology 91, no. 7: 925–932. 10.1002/JPER.19-0135. 31833563

[jcpe14149-bib-0026] Liberati, A. , D. G. Altman , J. Tetzlaff , et al. 2009. “The PRISMA Statement for Reporting Systematic Reviews and Meta‐Analyses of Studies That Evaluate Health Care Interventions: Explanation and Elaboration.” Journal of Clinical Epidemiology 62, no. 10: e1–e34. 10.1016/j.jclinepi.2009.06.006. 19631507

[jcpe14149-bib-0027] Lopez, N. J. , L. Jara , and C. Y. Valenzuela . 2005. “Association of Interleukin‐1 Polymorphisms With Periodontal Disease.” Journal of Periodontology 76, no. 2: 234–243. 10.1902/jop.2005.76.2.234. 15974847

[jcpe14149-bib-0061] Manolio, T. A. , F. S. Collins , N. J. Cox , et al. 2009. “Finding the Missing Heritability of Complex Diseases.” Nature 461, no. 7265: 747–753. 10.1038/nature08494. 19812666 PMC2831613

[jcpe14149-bib-0028] Meyle, J. , and I. Chapple . 2015. “Molecular Aspects of the Pathogenesis of Periodontitis.” Periodontology 2000 69, no. 1: 7–17. 10.1111/prd.12104. 26252398

[jcpe14149-bib-0029] Moher, D. , A. Liberati , J. Tetzlaff , D. G. Altman , and Group, P . 2009. “Preferred Reporting Items for Systematic Reviews and Meta‐Analyses: The PRISMA Statement.” Journal of Clinical Epidemiology 62, no. 10: 1006–1012. 10.1016/j.jclinepi.2009.06.005. 19631508

[jcpe14149-bib-0030] Molitor, A. , T. Prud'homme , Z. Miao , et al. 2019. “Exome Sequencing Identifies a Novel Missense Variant in CTSC Causing Nonsyndromic Aggressive Periodontitis.” Journal of Human Genetics 64, no. 7: 689–694. 10.1038/s10038-019-0615-3. 31068678

[jcpe14149-bib-0031] Morelli, T. , C. S. Agler , and K. Divaris . 2020. “Genomics of Periodontal Disease and Tooth Morbidity.” Periodontology 2000 82, no. 1: 143–156. 10.1111/prd.12320. 31850632 PMC6972532

[jcpe14149-bib-0032] Munz, M. , H. Chen , Y. Jockel‐Schneider , et al. 2017. “A Haplotype Block Downstream of Plasminogen Is Associated With Chronic and Aggressive Periodontitis.” Journal of Clinical Periodontology 44, no. 10: 962–970. 10.1111/jcpe.12749. 28548211

[jcpe14149-bib-0033] Munz, M. , C. Willenborg , G. M. Richter , et al. 2018. “A Genome‐Wide Association Study Identifies Nucleotide Variants at SIGLEC5 and DEFA1A3 as Risk Loci for Periodontitis.” Human Molecular Genetics 27, no. 5: 941–942. 10.1093/hmg/ddy015. 29346566

[jcpe14149-bib-0034] Nemeth, T. , K. Futosi , M. Szabo , et al. 2019. “Importance of Fc Receptor Gamma‐Chain ITAM Tyrosines in Neutrophil Activation and In Vivo Autoimmune Arthritis.” Frontiers in Immunology 10: 252. 10.3389/fimmu.2019.00252. 30858848 PMC6397848

[jcpe14149-bib-0035] Nibali, L. 2013. “Suggested Guidelines for Systematic Reviews of Periodontal Genetic Association Studies.” Journal of Clinical Periodontology 40, no. 8: 753–756. 10.1111/jcpe.12128. 23751179

[jcpe14149-bib-0036] Nibali, L. , J. Bayliss‐Chapman , S. A. Almofareh , Y. Zhou , K. Divaris , and A. R. Vieira . 2019. “What Is the Heritability of Periodontitis? A Systematic Review.” Journal of Dental Research 98, no. 6: 632–641. 10.1177/0022034519842510. 31107142 PMC6535921

[jcpe14149-bib-0037] Nibali, L. , G. Pelekos , F. D'Aiuto , et al. 2013. “Influence of IL‐6 Haplotypes on Clinical and Inflammatory Response in Aggressive Periodontitis.” Clinical Oral Investigations 17, no. 4: 1235–1242. 10.1007/s00784-012-0804-3. 22918663

[jcpe14149-bib-0038] Nishida, N. , M. Tanaka , S. Sekine , et al. 2010. “Association of ALDH2 Genotypes With Periodontitis Progression.” Journal of Dental Research 89, no. 2: 138–142. 10.1177/0022034509356045. 20042735

[jcpe14149-bib-0039] Noack, B. , H. Gorgens , U. Hempel , et al. 2008. “Cathepsin C Gene Variants in Aggressive Periodontitis.” Journal of Dental Research 87, no. 10: 958–963. 10.1177/154405910808701017. 18809751

[jcpe14149-bib-0040] Palmer, R. M. , R. F. Wilson , A. S. Hasan , and D. A. Scott . 2005. “Mechanisms of Action of Environmental Factors – Tobacco Smoking.” Journal of Clinical Periodontology 32, no. Suppl 6: 180–195. 10.1111/j.1600-051X.2005.00786.x. 16128837

[jcpe14149-bib-0041] Papapanou, P. N. , M. Sanz , N. Buduneli , et al. 2018. “Periodontitis: Consensus Report of Workgroup 2 of the 2017 World Workshop on the Classification of Periodontal and Peri‐Implant Diseases and Conditions.” Journal of Clinical Periodontology 45, no. S20: S162–S170. 10.1111/jcpe.12946. 29926490

[jcpe14149-bib-0042] Richter, G. M. , G. Wagner , K. Reichenmiller , et al. 2022. “Exome Sequencing of 5 Families With Severe Early‐Onset Periodontitis.” Journal of Dental Research 101, no. 2: 151–157. 10.1177/00220345211029266. 34515563 PMC8807999

[jcpe14149-bib-0043] Ritchie, S. C. , H. J. Taylor , Y. Liang , et al. 2024. “Integrated Clinical Risk Prediction of Type 2 Diabetes With a Multifactorial Polygenic Risk Score.” *medRxiv*. 10.1101/2024.08.22.24312440.

[jcpe14149-bib-0044] Roberts, E. , S. Howell , and D. G. Evans . 2023. “Polygenic Risk Scores and Breast Cancer Risk Prediction.” Breast 67: 71–77. 10.1016/j.breast.2023.01.003. 36646003 PMC9982311

[jcpe14149-bib-0045] Scapoli, C. , E. Mamolini , and L. Trombelli . 2007. “Role of IL‐6, TNF‐A and LT‐A Variants in the Modulation of the Clinical Expression of Plaque‐Induced Gingivitis.” Journal of Clinical Periodontology 34, no. 12: 1031–1038. 10.1111/j.1600-051X.2007.01145.x. 17953695

[jcpe14149-bib-0046] Schaefer, A. S. 2018. “Genetics of Periodontitis: Discovery, Biology, and Clinical Impact.” Periodontology 2000 78, no. 1: 162–173. 10.1111/prd.12232. 30198130

[jcpe14149-bib-0070] Schaefer, A. S. , L. Nibali , N. Zoheir , N. M Moutsopoulos , and B. G. Loos . 2025. “Genetic risk variants implicate impaired maintenance and repair of periodontal tissues as causal for periodontitis‐A synthesis of recent findings.” Periodontol 2000. https://doi/10.1111/prd.12622. Online ahead of print. 10.1111/prd.12622 PMC12350759 39953674

[jcpe14149-bib-0047] Schaefer, A. S. , G. M. Richter , M. Nothnagel , et al. 2010. “A 3' UTR Transition Within DEFB1 Is Associated With Chronic and Aggressive Periodontitis.” Genes and Immunity 11, no. 1: 45–54. 10.1038/gene.2009.75. 19829306

[jcpe14149-bib-0048] Shungin, D. , S. Haworth , K. Divaris , et al. 2019. “Genome‐Wide Analysis of Dental Caries and Periodontitis Combining Clinical and Self‐Reported Data.” Nature Communications 10, no. 1: 2773. 10.1038/s41467-019-10630-1. PMC6591304 31235808

[jcpe14149-bib-0049] Tada, H. , O. Melander , J. Z. Louie , et al. 2016. “Risk Prediction by Genetic Risk Scores for Coronary Heart Disease Is Independent of Self‐Reported Family History.” European Heart Journal 37, no. 6: 561–567. 10.1093/eurheartj/ehv462. 26392438 PMC4744619

[jcpe14149-bib-0050] Tikkanen, E. , A. S. Havulinna , A. Palotie , V. Salomaa , and S. Ripatti . 2013. “Genetic Risk Prediction and a 2‐Stage Risk Screening Strategy for Coronary Heart Disease.” Arteriosclerosis, Thrombosis, and Vascular Biology 33, no. 9: 2261–2266. 10.1161/ATVBAHA.112.301120. 23599444 PMC4210840

[jcpe14149-bib-0051] Tonetti, M. S. , N. Claffey , and European Workshop in Periodontology Group, C . 2005. “Advances in the Progression of Periodontitis and Proposal of Definitions of a Periodontitis Case and Disease Progression for Use in Risk Factor Research. Group C Consensus Report of the 5th European Workshop in Periodontology.” Journal of Clinical Periodontology 32, no. Suppl 6: 210–213. 10.1111/j.1600-051X.2005.00822.x. 16128839

[jcpe14149-bib-0052] Tonetti, M. S. , H. Greenwell , and K. S. Kornman . 2018. “Staging and Grading of Periodontitis: Framework and Proposal of a New Classification and Case Definition.” Journal of Periodontology 89, no. 1: S159–S172. 10.1002/JPER.18-0006. 29926952

[jcpe14149-bib-0053] Tong, H. , Z. Wei , J. Yin , et al. 2019. “Genetic Susceptibility of Common Polymorphisms in NIN and SIGLEC5 to Chronic Periodontitis.” Scientific Reports 9, no. 1: 2088. 10.1038/s41598-019-38632-5. 30765789 PMC6376118

[jcpe14149-bib-0054] Vuchkovska, A. , D. G. Glanville , G. M. Scurti , et al. 2022. “Siglec‐5 Is an Inhibitory Immune Checkpoint Molecule for Human T Cells.” Immunology 166, no. 2: 238–248. 10.1111/imm.13470. 35290663 PMC11590682

[jcpe14149-bib-0055] Whiting, P. F. , A. W. Rutjes , M. E. Westwood , et al. 2011. “QUADAS‐2: A Revised Tool for the Quality Assessment of Diagnostic Accuracy Studies.” Annals of Internal Medicine 155, no. 8: 529–536. 10.7326/0003-4819-155-8-201110180-00009. 22007046

[jcpe14149-bib-0056] Wray, N. R. , M. E. Goddard , and P. M. Visscher . 2007. “Prediction of Individual Genetic Risk to Disease From Genome‐Wide Association Studies.” Genome Research 17, no. 10: 1520–1528. 10.1101/gr.6665407. 17785532 PMC1987352

[jcpe14149-bib-0057] Wray, N. R. , S. Ripke , M. Mattheisen , et al. 2018. “Genome‐Wide Association Analyses Identify 44 Risk Variants and Refine the Genetic Architecture of Major Depression.” Nature Genetics 50, no. 5: 668–681. 10.1038/s41588-018-0090-3. 29700475 PMC5934326

[jcpe14149-bib-0062] Xu, C. , I. Tachmazidou , K. Walter , et al. 2014. “Estimating Genome‐Wide Significance for Whole‐Genome Sequencing Studies.” Genetic Epidemiology 38, no. 4: 281–290. 10.1002/gepi.21797. 24676807 PMC4489336

[jcpe14149-bib-0058] Yang, T. , B. Cheng , J. M. Noble , C. Reitz , and P. N. Papapanou . 2022. “Replication of Gene Polymorphisms Associated With Periodontitis‐Related Traits in an Elderly Cohort: The Washington Heights/Inwood Community Aging Project Ancillary Study of Oral Health.” Journal of Clinical Periodontology 49, no. 5: 414–427. 10.1111/jcpe.13605. 35179257 PMC9012699

[jcpe14149-bib-0059] Zabad, S. , S. Gravel , and Y. Li . 2023. “Fast and Accurate Bayesian Polygenic Risk Modeling With Variational Inference.” American Journal of Human Genetics 110, no. 5: 741–761. 10.1016/j.ajhg.2023.03.009. 37030289 PMC10183379

