# Supplementary material for: Genetic Biomarkers for Periodontal Diseases: A Systematic Review
Source: J Clin Periodontol. 2025 Apr 8;52(Suppl 29):182–210. doi: 10.1111/jcpe.14149 (PMC12286649; doi:10.1111/jcpe.14149)
Supplement: Supplementary file 1 — Data S1. [file JCPE-52-182-s001.docx]

**SUPPLEMENTAL MATERIAL 1**- SEARCH STRATEGY

**PubMed Search**

1. "periodontitis"[MeSH Terms] OR
2. "gingivitis"[MeSH Terms] OR
3. "periodontitis"[Text Word] OR
4. "gingivitis"[Text Word] AND
5. "genetic testing"[MeSH Terms] OR
6. "genetic test*"[Title/Abstract] OR
7. "genetic variant*"[Title/Abstract] OR
8. "genetic profile*"[Title/Abstract] OR
9. "epigenetic test*"[Title/Abstract] OR
10. "epigenetic variant*"[Title/Abstract] OR
11. "epigenetic profile*"[Title/Abstract] OR
12. "biomarker*"[Title/Abstract] OR
13. "marker*"[Title/Abstract]

**Search:** ((("genetic testing"[MeSH Terms] OR "genetic test*"[Title/Abstract] OR "genetic variant*"[Title/Abstract] OR "genetic profile*"[Title/Abstract] OR "genetic biomarker*"[Title/Abstract] OR "genetic marker*"[Title/Abstract] AND (("periodontitis"[MeSH Terms] OR "gingivitis"[MeSH Terms] OR "periodontitis"[Text Word] OR "gingivitis"[Text Word])))

**Ovid -Search**

**Link:**

**Database:**
Embase Classic+Embase <1947 to 2024 July 15>

**Search queries**

periodontitis.ab,ti.

gingivitis.ab,ti.

genetic test$.ab,ti.

genetic variant$.ab,ti.

genetic profile$.ab,ti.

genetic biomarker$.ab,ti.

genetic marker$.ab,ti.

**SUPPLEMENTAL MATERIAL 2-** Additional search criteria for genetic studies - not restricted to diagnostic measures (in PubMed)

**Search:** (((((periodontitis[MeSH Terms]) OR (gingivitis[MeSH Terms])) OR (periodontitis)) OR (gingivitis)) AND (genetic)) AND (polymorphism)

**Additional exclusion criteria:**

General:

- GWAS

For candidate gene studies on periodontitis diagnosis:

- studies reporting on a population with <1000 individuals (including test and control groups)

For candidate gene studies on periodontitis progression:

- studies reporting on <10 individuals

For candidate gene studies on gingivitis:

- studies reporting on <10 individuals

**Search result:** A total of 1592 papers were identified, of which 597 were included for further evaluation. Based on the above phrased exclusion criteria, 16 papers were included, and if possible, sensitivity and specificity for individual candidate gene testing was calculated by the reviewers.

**Supplementary figure 1**

Results for additional search for genetic studies not restricted to diagnostic measures (in PubMed).

**SUPPLEMENTAL MATERIAL 3-** Additional search criteria for candidate gene studies replicating gene variants detected in GWAS (in PubMed)

 ((((((periodontitis[MeSH Terms]) OR (periodontitis[Text Word])) OR (gingivitis[Text Word])) OR (gingivitis[MeSH Terms]))) AND (((((genetic validation) OR (genetic replication)) OR (GWAS)) OR (candidate gene))))

**Search result:** The additional search for validation studies revealed 745 entries, resulting in 7 suitable papers after first screening and 6 after full text screening (Cirelli, Nepomuceno, Goveia, et al., 2021; Cirelli, Nepomuceno, Orrico, et al., 2021; Schaefer et al., 2013; Taiete et al., 2019; Yang, Cheng, Noble, Reitz, & Papapanou, 2022).

**Supplementary figure 2**

Results of additional search for candidate gene studies replicating gene variants detected in GWAS (in PubMed).

**SUPPLEMENTAL MATERIAL 4-** Data extracted from included papers (if reported):

- Authors
- Year of publication
- Funding
- Study design
- Number of cases and controls included
- Age of included cases/controls
- Setting (country, hospital, university, community, other)
- Participants’ demographics (age, gender, ethnicity, smoking, socio-economic factors)
- Definition and diagnosis of periodontitis and healthy/reference controls (based on clinical measurements and radiographic images)
- Description of periodontal therapy
- Test and control diagnostic/prognostic ‘intervention’
- Type of (epi)genetic analysis performed
- Results of (epi)genetic analysis
- Primary outcome: diagnostic/prognostic accuracy vs gold standard
- Probing pocket depth
- Clinical attachment loss (CAL)
- Alveolar bone loss
- Diagnostic test
- Sensitivity
- Specificity
- Positive predictive value
- Negative predictive value
- Area under curve
- Cost-effectiveness

**SUPPLEMENTAL MATERIAL 5-** Reasons for exclusion at second stage screening

- No diagnostic accuracy testing or no parameters available for the computation of sensitivity or specificity
- No specific information on specific genotypes/ haplotypes and its link to periodontitis
- no information on periodontal diagnosis
- a minimum of 1000 individuals (in total: test and control)
